# Low-molecular-mass labile metal pools in *Escherichia coli*: advances using chromatography and mass spectrometry

**DOI:** 10.1007/s00775-021-01864-w

**Published:** 2021-05-08

**Authors:** Hayley N. Brawley, Paul A. Lindahl

**Affiliations:** 1 Department of Chemistry, Texas A&M University, College Station, TX 77843-3255 USA; 2 Department of Biochemistry and Biophysics, Texas A&M University, College Station, TX 77843 USA

**Keywords:** Labile metal pools, Electrospray ionization mass spectrometry, Cytosol, Size-exclusion chromatography, Iron, Copper, Zinc, Manganese

## Abstract

Labile low-molecular-mass (LMM) transition metal complexes play essential roles in metal ion trafficking, regulation, and signalling in biological systems, yet their chemical identities remain largely unknown due to their rapid ligand-exchange rates and weak M–L bonds. Here, an *Escherichia coli* cytosol isolation procedure was developed that was devoid of detergents, strongly coordinating buffers, and EDTA. The interaction of the metal ions from these complexes with a SEC column was minimized by pre-loading the column with ^67^ZnSO_4_ and then monitoring ^66^Zn and other metals by inductively coupled plasma mass spectrometry (ICP-MS) when investigating cytosolic ultrafiltration flow-through-solutions (FTSs). Endogenous cytosolic salts suppressed ESI-MS signals, making the detection of metal complexes difficult. FTSs contained ca. 80 µM Fe, 15 µM Ni, 13 µM Zn, 10 µM Cu, and 1.4 µM Mn (after correcting for dilution during cytosol isolation). FTSs exhibited 2–5 Fe, at least 2 Ni, 2–5 Zn, 2–4 Cu, and at least 2 Mn species with apparent masses between 300 and 5000 Da. Fe(ATP), Fe(GSH), and Zn(GSH) standards were passed through the column to assess their presence in FTS. Major LMM sulfur- and phosphorus-containing species were identified. These included reduced and oxidized glutathione, methionine, cysteine, orthophosphate, and common mono- and di-nucleotides such as ATP, ADP, AMP, and NADH. FTSs from cells grown in media supplemented with one of these metal salts exhibited increased peak intensity for the supplemented metal indicating that the size of the labile metal pools in *E. coli* is sensitive to the concentration of nutrient metals.

**Supplementary Information:**

The online version contains supplementary material available at 10.1007/s00775-021-01864-w.

## Introduction

Transition metals have unique and exceptional catalytic properties which make them indispensable for life [1]. They are typically installed into the active sites of metalloenzymes where they orchestrate catalytic events, often involving substrate binding, electron transfer, and/or small-molecule activation. Ironically, the same properties that make them indispensable for life also make them dangerous. Many iron and copper complexes react with O_2_ or H_2_O_2_ (ala the Fenton reaction) to generate reactive oxygen species that damage DNA, membranes, proteins, and other essential cellular components [2, 3]. The mismetallation of zinc and manganese into protein sites designed for other metals is also problematic [4, 5]. For this reason, metal ion *trafficking*—the translocation of a metal from the plasma membrane where it enters the cell, to the site of installation into its “client” apo-protein, is not only critical for the cell’s survival but must take place in a manner that protects the cell from the free metal and avoids toxic side reactions [6, 7]. In many cases, metals are passed from one protein chaperone to the next [8], but in others, low-molecular-mass (LMM) metal complexes are likely involved [7]. Such labile metal complexes or pools also appear to be involved in metal ion homeostasis and signalling [9–13]. Metal-associated diseases often involve metal ion dysregulation, altered trafficking patterns, and/or increased oxidative damage [14, 15]. A more chemical-level understanding of labile metal trafficking would improve the understanding of such biological processes and lead to new strategies for treating metal-associated diseases.

Neither the exact chemical compositions of these trafficking metal complexes nor their cellular functions are established [7]. This knowledge gap is due, in large measure, to their lability—i.e. they possess ligands that exchange rapidly due to the inherent weakness of metal–ligand coordinate bonds. Ligand exchange rates can be slowed by increasing the denticity of the ligands, employing certain metal oxidation or spin states, or using particular donor atoms and coordination geometries. We hypothesize that the rate of lability has been adjusted, through evolutionary pressures, to be slow enough for such complexes to “hold together” during transit (to avoid arbitrary deleterious reactions) yet fast enough to release the metal efficiently to its client apo-protein. Such trafficking complexes are presumed to have nonproteinaceous ligands composed of metabolites possessing O, N, and/or S Lewis-basic donor atoms.

The most popular strategy for studying labile metal pools in cells is to expose intact cells to custom-designed fluorescence-based chelators [16–20]. These chelators enter cells and change their fluorescence properties upon binding labile metals. Quantifying these changes allows the size of labile metal pools to be quantified. This approach has the advantage of not disrupting intact cells. However, the chelator-based approach *destroys* the sought-after metal complexes, making it unlikely that this approach could ever be used to chemically identify metal trafficking complexes [2]. Moreover, chelators are not completely specific for a particular metal, much less for a particular metal complex, and different chelators and reaction conditions yield different estimates of the size and properties of labile metal pools [7].

We are developing a complementary approach to study labile metal complexes in which cells are disrupted and soluble lysates are passed through an ultrafiltration membrane [21–24]. The resulting flow-through-solution (FTS) is passed down a size-exclusion chromatography (SEC) column, and the eluate is sent to an online ICP-MS. The column employed here resolves species with molecular masses between ca. 100 and 7000 Da. One challenge of this LC–ICP-MS approach is that endogenous metal trafficking complexes might be altered during sample preparation or during migration through the column; the advantage is the potential for collecting and identifying endogenous metal trafficking complexes and ultimately for establishing cellular roles.

We have attempted to identify labile metal complexes in the cytosol of *Escherichia coli* but have encountered problems along the way. These include unwanted effects of a common chelator, unwanted secondary interactions of labile metals on the column, unwanted ligand-exchange reactions, and the unwanted suppression of ESI-MS signals due to salts present in the cytosol. Here, we describe our efforts to overcome these problems. Using the lessons learned, we then examined the labile metal content of the cytosol from *E. coli* and detected numerous LMM labile metal complexes. Although we have not established the chemical identity or cellular function of these complexes, we are closer to doing so than ever before.

## Experimental procedures

### Strains and growth conditions

A derivative of *E. coli* K-12, MG1655, was transformed with bacteriophage-containing plasmid pZa31mycR [25]. MG1655-pZa31mycR cells were cultured in 50 mL of M9 minimal media containing 0.4% (w/v) glucose, 10 µM natural-isotopic-abundance Fe^III^ citrate, and 1 mM chloramphenicol (Sigma-Aldrich) overnight at 37 °C with 200 rpm shaking. These and all other concentrations given in the text are final concentrations after mixing. We determined by ICP-MS that M9 media contained 0.9 ± 0.6 µM Fe, 4 ± 1 µM Zn, 0.09 ± 0.01 µM Mn and 1 ± 1 µM Cu. Once grown, cultures were transferred to 1.0 L of growth media. Ten independent 1.0 L batches of cells were grown and harvested at early (OD_600_ ~ 0.6), mid- (OD_600_ ~ 1), or late (OD_600_ ~ 2) exponential phase by centrifuging at 4000×*g* for 15 min. Pellets (ca. 5 g wet cells) were washed in high-purity trace-metal-free double distilled-deionized water (HPW) and re-centrifuged at the same speed for 10 min. Pellets were resuspended in 5.0 mL of 20 mM ammonium bicarbonate (Sigma-Aldrich) buffered at pH 7.2. The suspension was transferred to a 15 mL polypropylene falcon tube, quickly frozen in liquid N_2_, and stored at − 20 °C. Additional batches were grown in which the medium was supplemented with either 100 µM of natural-isotopic-abundance Fe^III^ citrate (*n* = 4), Zn acetate (*n* = 3), MnCl_2_ (*n* = 1), or 1 µM CuSO_4_ (*n* = 1); these cells were harvested in mid-exponential phase.

### Isolation of cytosol and FTS

Frozen cells were thawed at 37 °C with 100 rpm shaking for 30 min. The lysate was centrifuged at 10,000×*g* for 5 min, transferred to a new 15 mL Falcon tube, and incubated with 20 µL of 1.12 mg/mL DNase (Sigma-Aldrich) and 10 mM MgCl_2_ (Acros Organics) for 30 min at 37 °C with 100 rpm shaking. Following DNA hydrolysis, the lysate was centrifuged at 100000×*g* for 60 min with a Beckman Coulter SW 32 Ti rotor in an Optima L-90K Ultracentrifuge. The resulting supernatant, defined as the cytosol, was brought into a chilled anaerobic glove box (MBraun Labmaster 120, 1–10 ppm O_2_, 4–8 °C) and passed through an Ultracel regenerated cellulose 3 kDa ultrafiltration disc (EMD Millipore) using an Amicon filtration system; the solution that passed through the membrane was defined as flow-through-solution (FTS).

### Metal and ligand standards

Stock solutions of Fe^II^ sulfate (Fisher chemical), Zn acetate (Acros Organics), MnCl_2_ (Sigma-Aldrich), CuSO_4_ (Acros Organics), and NiSO_4_ (Sigma-Aldrich) (1.0 mM each) were prepared in HPW. Similar stock solutions of reduced glutathione, GSH, (Sigma-Aldrich), oxidized glutathione, GSSG, (Sigma-Aldrich), cysteine (Sigma-Aldrich), methionine (MP Biomedicals), Na_2_HPO_4_ (Sigma-Aldrich), NaH_3_P_2_O_7_ (Sigma-Aldrich), Na_4_NADPH (Sigma-Aldrich), Na_2_NADH (Sigma-Aldrich, Na_2_AMP (Sigma-Aldrich), NaADP (Sigma-Aldrich), Na_2_ATP (Sigma-Aldrich) were prepared in HPW. Additional 10 mM and 100 mM stocks of GSH and ATP were prepared in HPW. All stocks were stored at 4 °C. For LC–ICP-MS analysis, stock standards were diluted (day-of) to the desired final concentration in mobile phase (see LC–ICP-MS and elemental analysis). Na(polyphosphate) (Sigma-Aldrich) was prepared at 1.1 g/mL and filtered through the 3 kDa membrane; the filtrate was diluted in mobile phase prior to LC–ICP-MS analysis.

### LC–ICP-MS and elemental analysis

Primary LC–ICP-MS analyses were performed on a single Superdex™ Peptide 10/300 GL (GE Life Sciences) SEC column. Additional analyses were performed on two such columns linked in series. The mobile phase passed through the column at 0.6 mL/min for the single and 0.25 mL/min for the double column using an Agilent 1260 bio-inert quaternary pump (G5611A) with diode array (G4212B), fraction collector (G5664A), and multisampler (G5688A). The entire LC system was located inside the glove box. Eluate flowed to an ICP-MS (Agilent 7700x) located outside of the box where ^23^Na, ^39^K, ^31^P, ^34^S, ^55^Mn, ^56^Fe, ^57^Fe, ^60^Ni, ^63^Cu, ^65^Cu, ^66^Zn, ^67^Zn, and ^68^Zn were detected. The mobile phase was either 20 mM or 50 mM ammonium acetate (AA) pH 6.5 for LC–MS (Sigma-Aldrich), which had previously passed through a 0.22 µm filter using a Stericup vacuum filtration system (Corning) and then degassed using a Schlenk line prior to import into the box. AA was selected as the mobile phase buffer due to its volatility and compatibility with both ICP-MS and ESI-MS. Samples (150 µL) were injected automatically using the multisampler. Peak elution volumes (*V*_*e*_) were calibrated to molecular masses using standards listed in Table S1. Designated peaks were simulated with Fityk software (fityk.nieto.pl) employing the Levenberg–Marquardt algorithm with a built-in Gaussian function.

For elemental analysis of samples, three aliquots (50–100 µL) of lysate, cytosol, and FTS from the ten independent batches were transferred into 15 mL polypropylene falcon tubes. 150 µL of trace-metal-grade 70% (w/v) nitric acid (Thermo Fisher Scientific) was added to each tube. Tubes were capped, sealed with electrical tape, vortexed, and incubated at 70 °C for ~ 15 h. Samples were cooled to RT and then diluted to a final volume of 3 mL with HPW followed by ICP-MS analysis.

### ^67^Zn loading of SEC column

Prior to loading, the column was cleaned by passing 500 µL of a chelator cocktail through it. The cocktail included 50 µM each of ethylenediaminetetraacetic acid (EDTA) (Sigma-Aldrich), ethylene glycol-bis(β-aminoethyl ether)-*N*,*N*,*N*′,*N*′-tetraacetic acid (EGTA) (Sigma-Aldrich), 1,10-phenanthroline (phen) (Acros Organics), 2,2-bipyridine (BPY) (Alfa Aesar), bathocuproinedisulfonic acid (Sigma-Aldrich), deferoxamine (END Millipore), (*N*,*N*,*N*′,*N*′-tetrakis(2-pyridinylmethyl)-1,2-ethanediamine (TPEN) (Sigma-Aldrich), and 1 mM ascorbic acid (Acros Organics). Three separate aliquots of the cocktail were injected onto the column with alternating injections of 500 µL HPW. A column volume (CV = 24 mL) of the mobile phase 50 mM AA (Sigma-Aldrich), pH 6.5, was passed between injections.

Three methods were used to load the column. In the first method, > 10 CVs of 10 µM ^67^ZnSO_4_ (90%; Isoflex USA) in HPW was passed as the mobile phase through the column, and then the mobile phase was changed to 50 mM AA, pH 6.5, to rinse-off unbound ^67^Zn. This method eventually contaminated the LC system with ^67^Zn, which was painstakingly removed by excessive flushing with dilute HCl, pH 3. In the second method, 500 µL of 10 µM ^67^ZnSO_4_ was injected onto the column. After passing 1 CV of 50 mM AA, pH 6.5, mobile phase, 500 µL of HPW was injected, followed by another CV of mobile phase. These injections were repeated 4 × more. The column was then rinsed with 50 mM AA, pH 6.5, until a flat baseline for ^66^Zn was attained. In the third method (currently used), 5 CVs of a mobile phase consisting of 5 µM ^67^ZnSO_4_ in 50 mM AA, pH 6.5, were passed through the column, followed by 50 mM AA, pH 6.5, until a flat ^66^Zn baseline was achieved. Due to heavy use, the loading procedure was repeated bimonthly.

### ESI-MS analysis

Electrospray ionization mass spectrometry (ESI-MS) was performed using a Thermo Scientific Q Exactive Focus (Waltham, Massachusetts) instrument. FTSs, LC fractions, and standards (GSH, ATP, etc.) were diluted 2 × or 20 × (5 µL sample + 5 µL CH_3_OH for 2 × ; 10 µL sample + 200 µL CH_3_OH for 20 × using LC–MS-grade methanol (Thermo Fisher Scientific)), depending on metal and salt concentrations in the sample. Samples were injected into a 10 µL loop, using methanol as a mobile phase at a flow rate of 300 µL/min. The Q Exactive Focus HESI source was operated in full MS (66–1000 m/z) in positive and negative modes. The mass resolution was tuned to 70,000 FWHM at *m*/*z* 200. Spray voltage was 3.5 kV for positive mode and 3.3 kV for negative mode. Sheath gas and auxiliary gas flow rates were 7 and 0 AU, respectively. Transfer capillary temperature was held at 270 °C and the S-Lens RF level was 50 V. Exactive Series 2.11/Xcalibur 4.2 software was used for data acquisition and processing. Mass accuracy was within ± 2 ppm.

## Results

Our long-term objective is to determine the chemical composition of the LMM labile metal pools in *E. coli* and other biological systems. We previously detected LMM metal complexes in *E. coli* and *Saccharomyces cerevisiae* [21–24] but did not identify them; the main objective of those studies was to establish reproducibility. We initially focussed on zinc because of its redox inactivity and ability to form stable coordination complexes, properties that increased our likelihood of success. We selected the Gram-negative model bacterium *E. coli* because much supporting mechanistic information was known about metal ion metabolism in this organism. FTS, which should exclusively contain species with masses < 3 kDa, was subjected to SEC. Eluates were sent directly to an ICP-MS for the detection of metals, sulfur, and phosphorus. The LC was located in a refrigerated inert atmosphere glove box to avoid oxidation of metal ions and sulfhydryl groups and to minimize ligand-exchange reactions.

### A holin/endolysin-containing strain allowed cell lysis without EDTA

**Fig. 1:**
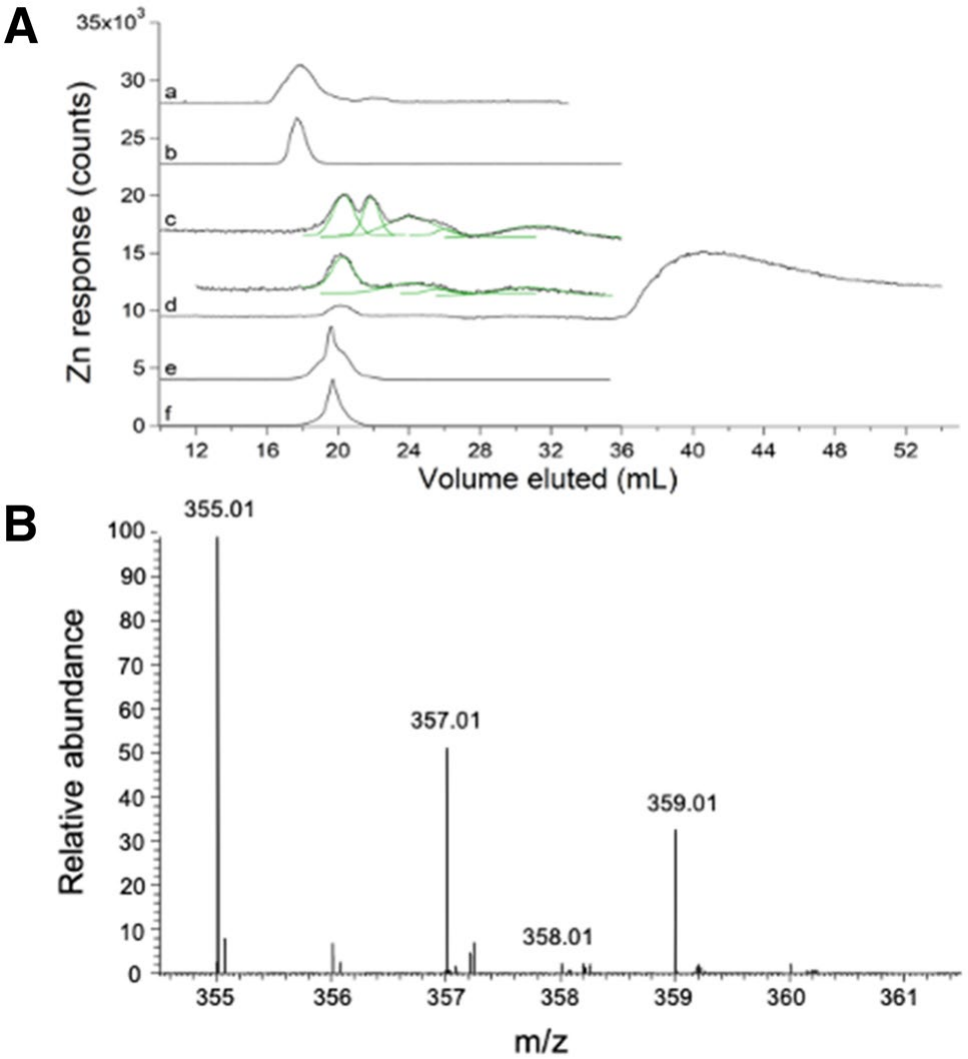
Zn-detected LC–ICP-MS chromatograms (**A**) and ESI-MS spectrum (**B**) of *E. coli* FTS: **A**: (a) average of 3 traces of cytosolic FTS isolated from MG1655 *E. coli* cells using EDTA [21]. (b) 1 µM ZnCl_2_ + 1 µM EDTA ÷ 4. The mobile phase for (a) and (b) was 20 mM ammonium bicarbonate pH 8.5. (c) average of 8 independent traces of FTS from RYMG1655 + pZa31mycR *E. coli* cytosol (black) overlaid with simulations (green). Unless specified otherwise, no EDTA was used during isolation, the default mobile phase was 50 mM AA pH 6.5, and the cells were MG1655 + pZa31mycR. (d) Average of 2 traces from independent FTSs of cells grown in 100 µM Zn supplemented growth medium. Offset is (d) × 5. (e), FTS as in (c) but incubated with 500 µM EDTA ÷ 20. (f), 1 µM Zn acetate + 20 µM EDTA ÷ 10. **B:** Positive mode spectra of Zn-containing LC fractions from FTSs isolated as in (a), then lyophilized and resuspended in D_2_O. Lines with indicated masses reflect the 1 + charge state of Zn(EDTA) and showed the expected isotope pattern. × # and ÷ # refer to an #-fold multiplication/division of the detector response in the plotted trace

We became acutely concerned with the chelator EDTA in our buffers when we noticed that the LMM Zn species previously detected in FTSs (Fig. 1A, trace a) co-migrated with Zn(EDTA) (Fig. 1A, trace b). To investigate further, we lyophilized the Zn-containing fractions that eluted from the column and rehydrated the dried material in minimal D_2_O. ESI-MS of the resulting solution (Fig. 1B) demonstrated the presence of Zn(EDTA), including the pattern expected from the natural isotope distribution (49% ^64^Zn; 28% ^66^Zn; 4% ^67^Zn; 18% ^68^Zn). We removed EDTA from all buffers but discovered that removing it from the lysis buffer decreased the effectiveness of cell lysis. We switched to a custom strain of *E. coli* (MG1655 + pZa31mycR) in which lysis occurred via canonical phage lysis. The strain contained a plasmid encoding two phage proteins, holin and endolysin, which upon freezing and thawing initiated cell lysis. This strain was used for the remainder of the study.

### EDTA-free cytosolic FTS contained 2–5 labile LMM zinc species with a collective concentration of ~ 13 µM

We isolated cytosolic FTSs from ten independent batches of *E. coli* cells in the absence of EDTA and passed them through a ^67^Zn-loaded column (see below). The cells contained an average of 400 ± 200 µM Zn, whereas isolated cytosol contained 200 ± 100 µM Zn, and FTS contained 13 ± 3 µM Zn (determinations were back-calculated to concentrations within the cell). EDTA-free FTS displayed a different chromatogram that included, on average, 5 partially resolved LMM Zn peaks (Fig. 1A, trace c). The green lines simulating these peaks used parameters given in Table S2. A few individual traces exhibited just 2 of those Zn peaks (Fig. S1). We considered that the growth phase at harvest (early, mid, or late exponential) might reveal significant differences, but none were evident and so traces were averaged. Traces were obtained using 50 mM AA, pH 6.5, the default mobile phase for the entire study. When EDTA-free FTS was treated with EDTA, the major peak in the resulting trace (Fig. 1A, trace e) migrated with Zn(EDTA) (Fig. 1A, trace f). Clearly, Zn peaks obtained in the absence of EDTA more accurately represented the labile zinc pool in *E. coli*. Under these growth conditions, this pool constituted about 3% of the total zinc in the cell. We concluded that the detected Zn species were labile towards EDTA, and that the LMM Zn complex previously reported [21] was probably Zn(EDTA). All other FTSs described in this manuscript were isolated in the absence of EDTA.

Supplementing the growth medium with 100 µM Zn acetate led to large increases in the LMM Zn pool, to an average of 200 ± 100 µM (range from 70 to 320 µM). Most of the additional Zn eluted as an intense broad peak at ca. 40 mL (Fig. 1A, trace d) which likely arose from hydrated Zn ions that interacted strongly with the column. Such peaks were only observed when the growth medium was supplemented with Zn acetate. Curiously, the Zn peak with *V*_*e*_ ≈ 21 mL in traces from un-supplemented FTS was absent in supplemented FTS whereas the other 4 peaks were present under both conditions, and with similar relative intensities. See Table S2 for parameters used to simulate these and other peaks.

### Zinc loading minimized metal interaction with columns

We previously reported that labile LMM metal complexes partially adsorbed onto, and desorbed from, the SEC column [22, 23]. Metal ions likely participated in secondary ionic interactions with basic groups on the solid support such as carboxylates [26]. Previously, we cleaned the column regularly and extensively using a chelator cocktail, and on occasion, with dilute acid and base. However, low detector response and spurious metal peaks remained problematic. The latter effect was due to “injection-initiated” metal ion desorption in which simply injecting a sample perturbed the column sufficiently to dislodge small quantities of metal ions.

We developed three methods to minimize adsorption/desorption problems further, all of which involved saturating basic sites on the column with a particular isotope of zinc (^67^Zn) and then detecting two different isotopes (^66^Zn and ^68^Zn) in subsequent analyses of samples containing natural-abundance isotopes of zinc. Zinc loading by Method 2 (see “Experimental procedures”) caused ~ 0.1% of the sites on the solid support to coordinate ^67^Zn ions. Loading by Method 3 caused binding of ~ 2.5% of sites. Although fewer sites were bound by Method 2, blocking them was enough to minimize the interaction of hydrated metal ions with the column. We suspect that the affinity of the sites to Zn was variable and that the strongest binders caused most of the problem. Method 3 provided the best reproducibility and largely eliminated spurious LC peaks. Regardless of the method, loaded ^67^Zn ions gradually desorbed, such that the column had to be reloaded periodically.

**Fig. 2:**
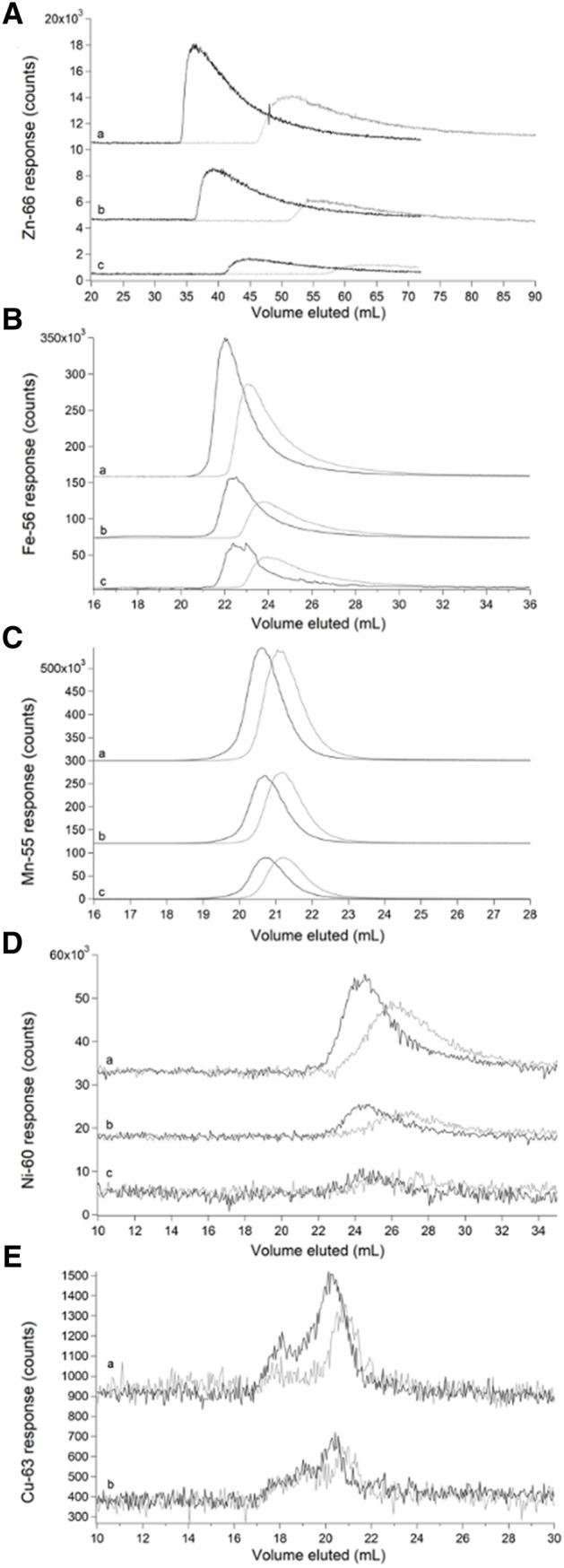
Chromatograms of aqueous zinc (**A**), iron (**B**), manganese (**C**), nickel (**D**), and copper (**E**) on an unloaded (grey) and ^67^ZnSO_4_-loaded single SEC column (black). **A** (a–c), 5, 2, and 1 µM Zn acetate, respectively. **B** (a–c), 5, 2, and 1 µM FeSO_4_. **C** (a–c), 5, 2, and 1 µM MnCl_2_. **D** (a–c), 5, 2, and 1 µM NiSO_4_. **E** (a–b), 5 and 1 µM CuSO_4_

The behavior of Zn loading is illustrated in Fig. 2. The grey ^66^Zn-detected traces in Fig. 2A, traces a–c were obtained by passing 5, 2, and 1 µM natural-abundance Zn acetate through an unloaded column. Peaks were extremely broad and showed severe tailing. Distorted peak shapes likely reflected binding interactions with the column that were strong enough to hinder passage but weak enough to allow passage within the timeframe of the experiment. As the concentration of Zn increased (c → a), the elution volume (*V*_*e*_), tailing, and peak-width all decreased. We concluded that as the concentration of Zn in the sample increased, column interactions declined, requiring less elution volume and affording greater homogeneity. The same phenomenon was evident in the corresponding black ^66^Zn traces of Fig. 2A, which were obtained by passing the same solutions through a ^67^Zn-loaded column. Elution volumes were reduced further when the loaded column was used, and linewidths were narrowed, indicating diminished column interactions. We used a “ghost column” (consisting of PEEK tubing that replaced the actual column) and peak-fitting software to show that > 90% of injected Zn in samples eventually eluted from *both* loaded and unloaded columns.

A similar though less severe phenomenon was evident by passing aqueous iron, nickel, and manganese ions through the column. The grey traces in Fig. 2B, obtained by passing 5, 2, and 1 µM FeSO_4_ through the column, exhibited broad peaks with some tailing. As the concentration of iron increased (c → a), *V*_*e*_, linewidths, and tailing all decreased. The corresponding black traces, obtained by injecting the sample solutions onto a loaded column, exhibited sharper peaks and lower *V*_*e*_. Again, this illustrated a decline in column interactions due to ^67^Zn loading. Nearly 100% of the injected iron eventually eluted from the column regardless of whether it was loaded. Two effects might have been involved, including an interaction with the column that delayed elution and caused tailing, and an “overloading” effect in which disproportionately more metal ions bound to the column when higher concentrations were injected, thereby causing injector-initiated spurious metal peaks. The same trend was observed when passing 5, 2, and 1 µM NiSO_4_ through the columns (Fig. 2D). Passing manganese ions through the column afforded sharp and nearly Gaussian peaks (Fig. 2C) regardless of whether the column was loaded. Of the metals tested, manganese ions probably interacted least with the column.

The opposite situation was found with aqueous copper ions, as little of the injected copper eluted from the column (Fig. 2E). More copper eluted from the ^67^Zn-loaded column than from an unloaded column, but peak shapes were similar. Only 1% of the copper injected onto the Zn-loaded column eluted; the rest must have been adsorbed, accounting for the poor signal-to-noise ratio. The observed signal is likely due to contamination in the mobile phase. We suspect that aqueous copper ions bound the column so strongly that they displaced bound ^67^Zn ions; however, we were unable to detect copper-dependent ^67^Zn elution. The different behaviors observed followed the Irving–Williams series [27] in which the order of binding strengths (to classical O-, N-, and S-based ligands) varied in the order (weakest) Mn^II^ < Fe^II^ < Ni^II^ < Zn^II^ < Cu^II^ (strongest).

## Chromatographic behavior of iron and zinc standards reflected the M–L binding strength of the complex

**Fig. 3:**
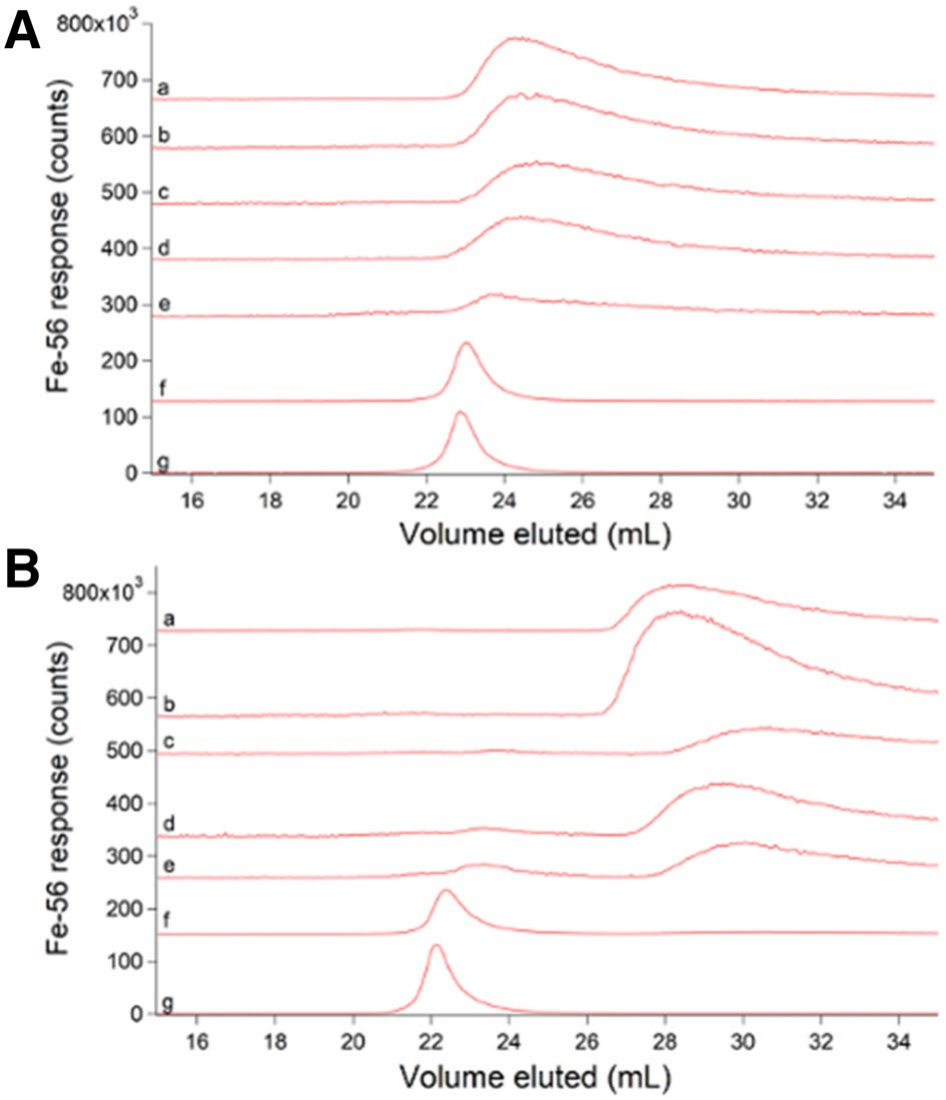
Iron-detected chromatograms of Fe(ATP) using 50 mM (**A**) and 20 mM (**B**) ammonium acetate pH 6.5 mobile phases. All traces were from samples containing 1 µM FeSO_4_ + the following (final) µM concentrations of Na_2_ATP. **A**: (a) 0; (b) 5; (c) 10; (d) 25; (e) 50; (f) 500; (g) 1000. **B**: (a) 0; (b) 5; (c)10; (d) 25; (e) 50; (f) 500; (g) 1000

At this point, we shifted focus to iron, as its interaction with the ^67^Zn-loaded column was weaker than zinc’s, yielding sharper peaks that were easier to study. Iron(ATP) is a candidate cytosol trafficking complex [28], and so we examined its chromatographic properties by mixing 1 µM FeSO_4_ with increasing concentrations of ATP. Both iron and phosphorus signals were monitored. In the absence of ATP, iron migrated as a broad tailing peak with *V*_*e*_ ≈ 24 mL (Fig. 3A, trace a). As ATP concentrations increased (and with [FeSO_4_] fixed at 1 µM), the iron peak shifted left and sharpened, ultimately eluting at ca. 23 mL. This behavior indicated that iron and ATP formed a complex at sufficiently high concentrations of ATP but that the complex dissociated as it migrated through the column such that the iron eluted at different mobile phase volumes depending on the ATP concentration. The chromatographic behavior of ATP was independent of iron but was unexpectedly complicated nevertheless (Fig. S2, panel A). ATP migrated as 3 peaks, including a major peak at 23.5 mL and two low-intensity “satellites” at 22 mL and 25.5 mL. The satellite peaks did not comigrate with inorganic phosphate or ADP, which would have indicated hydrolysis of the standard. They may have been due to impurities in the ATP standard.

The chromatographic behavior of Fe(ATP) changed when the concentration of AA in the mobile phase was lowered from 50 to 20 mM. Using 20 mM AA, the 1 µM FeSO_4_ sample lacking ATP eluted as a broad trailing iron peak at ca. 27 mL (Fig. 3B, trace a), a downstream shift of ca. 2 mL relative to the peak obtaining using 50 mM AA. We interpreted this as indicating a stronger interaction between aqueous iron and the column. As the ATP concentration increased, the broad tailing peak remained until the ATP concentration was ≥ 500 µM. At such high ATP concentrations, the broad tailing peak was replaced by a sharper peak with a more Gaussian lineshape ca. 23 mL (Fig. 3B, traces f, g). This peak shifted and sharpened with increasing ATP concentration. We concluded that an Fe(ATP) complex formed more tightly when 20 mM AA was used in the mobile phase but that the interaction between iron and the column was also stronger, giving rise to the broad tailing and lack of comigrating Fe and P signals. The corresponding phosphorus traces using 20 mM AA followed the same general trend as with 50 mM AA, including the two satellite peaks (Fig. S2, panel B).

**Fig. 4:**
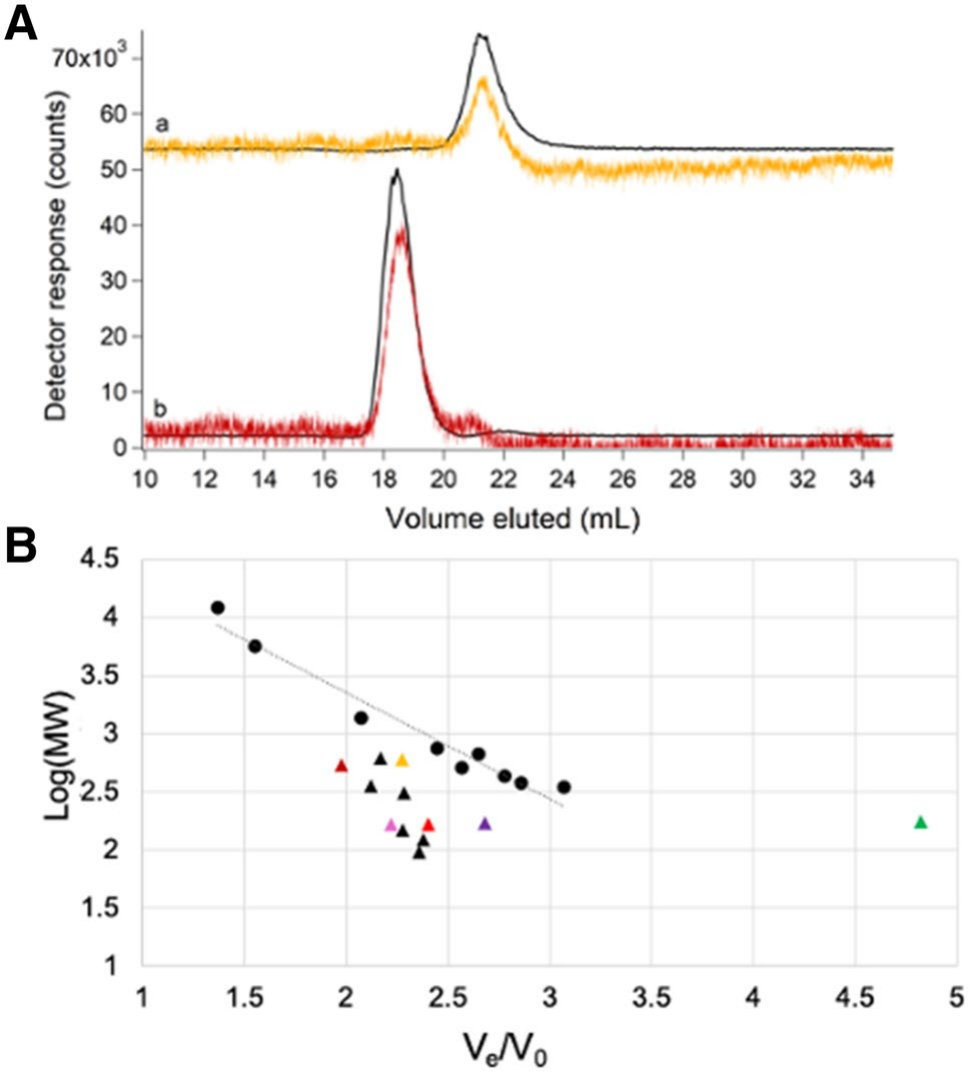
Chromatograms of [Fe(phen)_3_]^2+^ (orange) and [Fe(BPY)_3_]^2+^ (red) (**a**) and deviations from expected molecular mass trend line (**B**). **A**: (a**)** 2 µM FeSO_4_ + 20 µM phen detected by iron ICP-MS (black, ÷ 1.33) and at 510 nm (orange, × 10^4^); (b) 2 µM FeSO_4_ + 20 µM BPY detected by iron ICP-MS (black, ÷ 2) and at 523 nm (red, × 10^4^). **B**: Molecular mass calibration curve and trendline (log(MM) =  − 0.9204(*V*_e_*/V*_0_) + 5.1971; *R*^2^ = 0.9575) using standards from Table S1 (circles) and a Zn-loaded single column. Deviant standards are shown as triangles. [Fe(phen)_3_]^2+^ and [Fe(BPY)_3_]^2+^ are color-coordinated to UV–Vis traces in **A**. Aqueous metal standards were Zn acetate (green), FeSO_4_ (bright red), MnCl_2_ (pink) and NiSO_4_ (purple)

Traces of two tight-binding iron complexes, [Fe(phen)_3_]^2+^ and [Fe(BPY)_3_]^2+^, where phen = 1,10-phenanthroline and BPY = 2,2′-bipyridine, were simpler to interpret. Reported logβ values for these complexes are 21.2 and 17.5, respectively [29]. Our LC–ICP-MS system included an online diode-array UV–Vis spectrometer. This allowed the intact colored complexes and the metal to be monitored simultaneously and independently as they eluted from the column. Both iron and UV–Vis traces of [Fe(phen)_3_]^2+^ and [Fe(BPY)_3_]^2+^ exhibited single comigrating peaks (Fig. 4A, traces a, b), establishing that these complexes remained intact as they migrated through the column.

The column was calibrated by plotting the logarithm of the standard molecular mass *vs.* the ratio of *V*_*e*_ to void volume (*V*_e_/*V*_0_) where *V*_0_ was determined to be 9.3 mL using thyroglobulin. We expected that species would migrate through the column with *V*_*e*_ inversely proportional to the logarithm of molecular mass of the species, but this was not always the case (Fig. 4B, triangles). For example, [Fe(phen)_3_]^2+^ has a higher molecular mass than [Fe(BPY)_3_]^2+^ (570 vs. 524 Da), but it migrated as though it had a lower mass (Fig. 4B, yellow vs. dark red triangle). This problem was exacerbated for weakly coordinated metal complexes in which *V*_*e*_ shifted with changes in the concentration of the ligand, mobile phase, and the extent of interaction with the column.

**Fig. 5:**
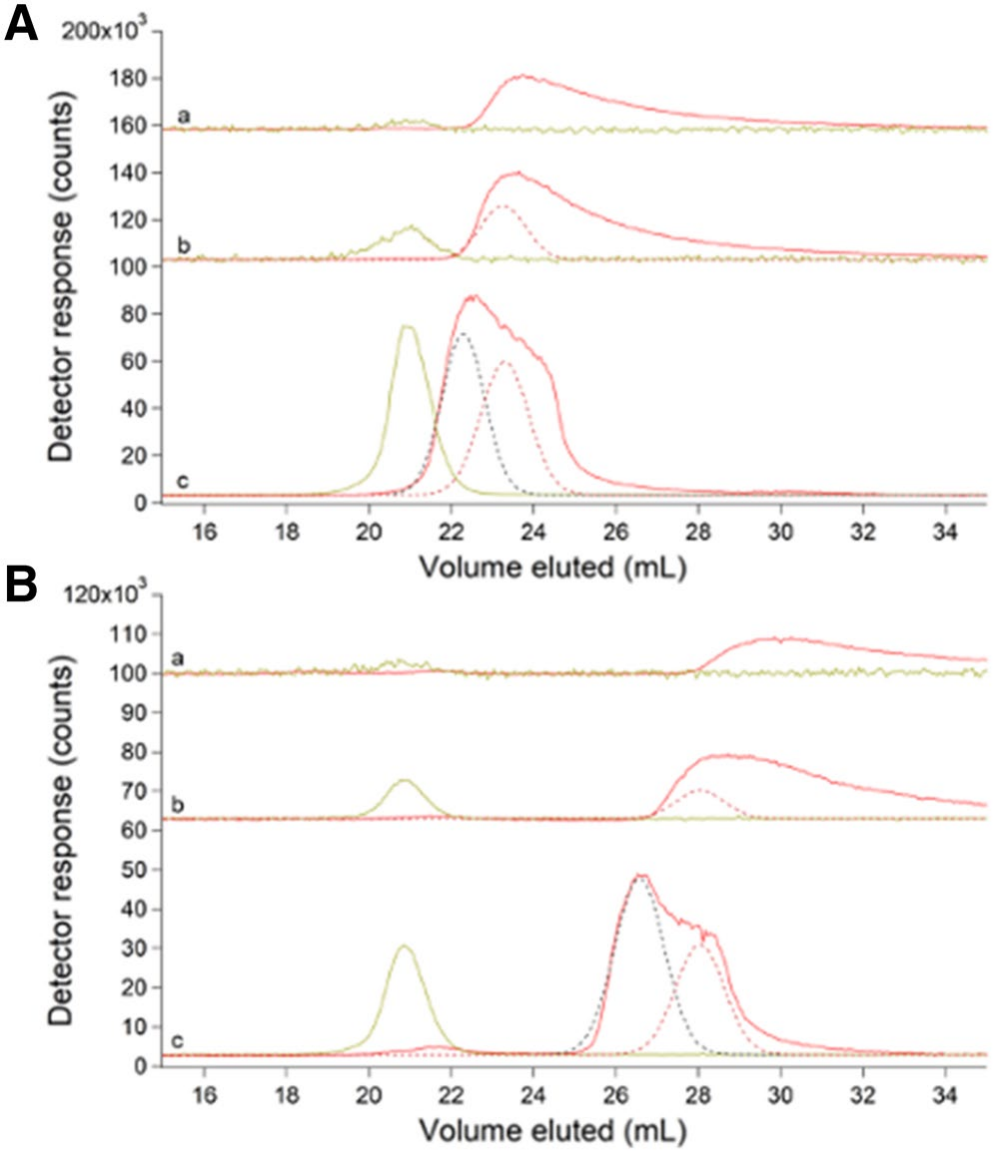
Chromatograms of FeSO_4_ (red) and GSH (yellow) using 50 mM (**A**) and 20 mM (**B**) AA mobile phase buffers. **A**: (a) 1 µM FeSO_4_ + 100 µM GSH × 5; (b) same as (a) but with 1 mM GSH; (c) same as (a) but with 100 mM GSH ÷ 10. **B**: (a) 1 µM FeSO_4_ + 100 µM GSH × 5; (b) same as (a) but with 1 mM GSH ÷ 10; (c) same as (a) but with 50 mM GSH ÷ 10. Dashed lines are simulations

Fe(GSH) is another candidate cytosol trafficking complex [30, 31], and so we examined the chromatographic properties of 1 µM FeSO_4_ solutions mixed with increasing concentrations of GSH in hopes of generating the complex. Each solution was passed down the column (using 50 mM AA mobile phase), and the eluate was monitored for iron and sulfur (Fig. 5A). The experiment was repeated using 20 mM AA mobile phase (Fig. 5B). GSH migrated with *V*_*e*_ ≈ 21 mL regardless of mobile phase whereas *V*_*e*_ for the iron peaks shifted depending on the GSH concentration and mobile phase. At low GSH concentrations, iron migrated as a broad tailing peak at *V*_*e*_ ≈ 23 mL (Fig. 5A, trace a) and 29 mL (Fig. 5B, trace a), depending on mobile phase. These peaks were nearly identical to those observed with FeSO_4_ alone. In solutions containing intermediate concentrations of GSH, the iron peak shifted left and sharpened (Fig. 5A, trace b and 5B, trace b). The traces of solutions containing 50 or 100 mM GSH had a broad peak that migrated at about the same *V*_*e*_ as peaks present in solutions containing 0.1 and 1 mM GSH (red dashed lines). In addition, the traces involving 50 or 100 mM GSH contained an intense Fe peak at ca. 22 mL (Fig. 5A, trace c) and at ca. 26 mL (Fig. 5B, trace c), simulated in the dashed black lines. Also, trace c of Fig. 5B exhibited a minor Fe peak at ca. 22 mL which partially overlapped the dominant sulfur peak centered at 21 mL. We regard both iron peaks (at 22 mL using 50 mM AA and 26 mL using 20 mM AA) as Fe(GSH) candidates. This behavior (shifting due to changing the concentration of the coordinating ligand, and development of new peaks when high concentrations of the ligand are used) indicated weaker binding for Fe(GSH) than for either [Fe(phen)_3_]^2+^ or [Fe(BPY)_3_]^2+^. Consistent with this assessment, reported stability constant for Fe(GSH) is logβ = 5.1–5.6 [30].

Despite zinc’s strong interaction with the ^67^Zn-loaded column, we assessed its chromatographic properties by mixing 2 µM Zn acetate solutions with either 0.1, 1, or 100 mM GSH (Fig. S3). Each solution was passed down the column (using 50 mM AA mobile phase), and the eluate was monitored for zinc and sulfur. Direct comigration of zinc and sulfur peaks at ca. 21 mL was only observed when GSH concentration was 100 mM (Fig. S3, trace D); complexation of Zn(GSH) was confirmed via positive-mode ESI-MS (*m*/*z* = 370.00, 372.00, 373.00, 374.00) for the fraction containing the standard peak. As with the Fe(GSH) standards, when increasing amounts of GSH were mixed with Zn acetate, a shift in the Zn trace was observed. This demonstrated weak binding of Zn(GSH).

### The LMM sulfur pool consisted of GSH, GSSG, methionine and cysteine

**Fig. 6:**
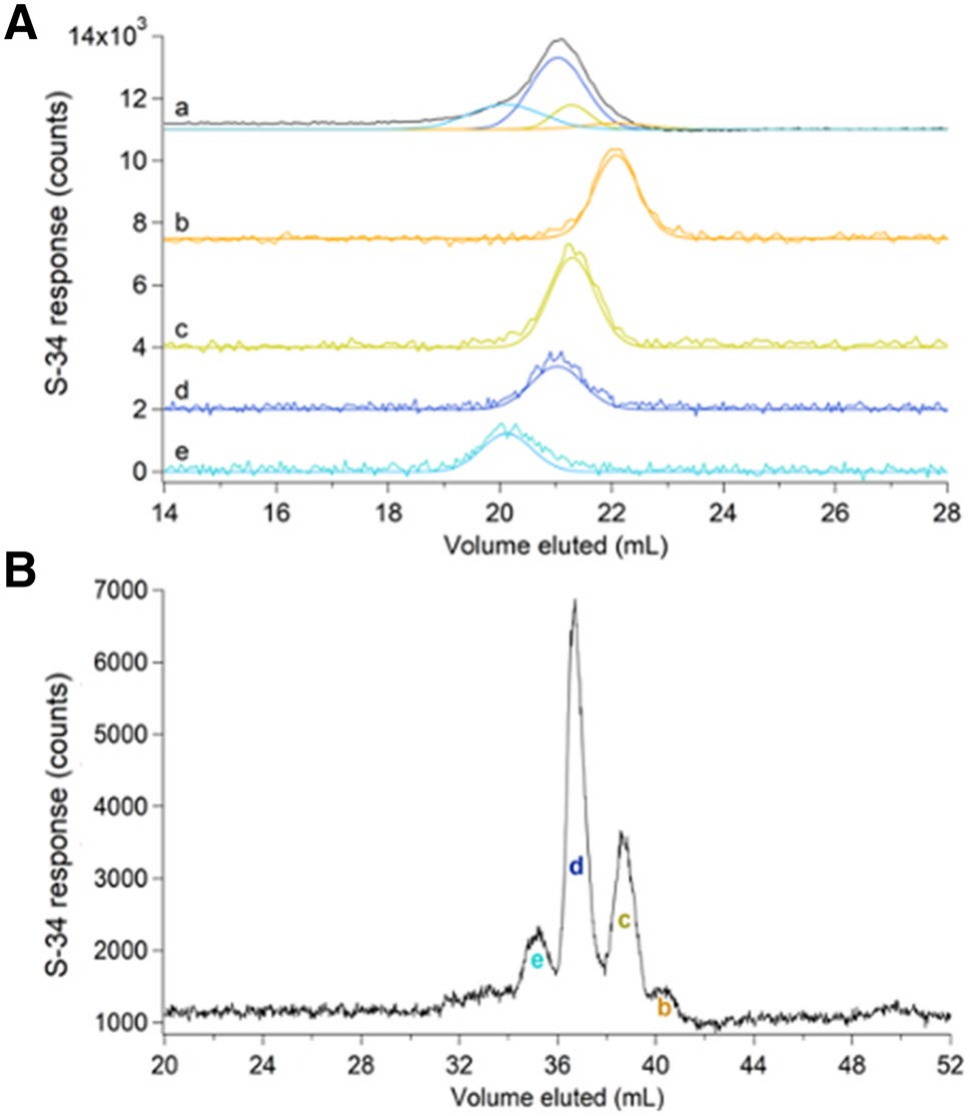
Sulfur-detected chromatograms of FTS and standards on the single (**A**) and double (**B**) column. **A**: (a), averaged FTS trace (black) and simulations (colored lines coded with standard simulations below). (b), 500 µM cysteine; (c), 500 µM methionine; (d), 250 µM GSH; (e), 250 µM GSSG. **B**: FTS replicate with peaks (b)–(e) correspond to standards in **A** that were identified by positive-mode ESI-MS

The averaged sulfur-detected trace of cytosolic FTSs exhibited a broad unresolved peak suggesting multiple contributing species (Fig. 6A, trace a). Individual sulfur traces of FTS are given in Fig. S4. Cysteine, methionine, GSH, and GSSG standards migrated in this region (Fig. 6A, traces b–e), suggesting that they might contribute to the observed broad FTS peak. Peaks from each species were simulated (color-coded lines in Fig. 6A, trace a) and combined to recreate the overall experimental trace (black line). To better resolve each contribution, FTS was passed through the double ^67^Zn-loaded column. Sulfur-detected traces exhibited 4 resolved species (Fig. 6B). The ESI-MS spectra of fractions collected when FTS eluted from the double column (Fig. S5) included peaks at *m*/*z* = 613.16, 308.09, 150.06, and 122.03 Da for LC peaks e, d, c, and b, respectively in Fig. 6B. This confirmed the presence of the GSSG, GSH, methionine, and cysteine as predicted by fitting the unresolved peak obtained with the single column. The intensity of each contributing simulation was converted into absolute concentration using calibrated intensities of standard peaks, and those concentrations were multiplied by the dilution factors involved in isolating FTS. Accordingly, the concentrations of GSH, GSSG, methionine, and cysteine in *E. coli* cytosol were calculated to be 3000, 400, 800, and 200 µM, respectively. The concentration for GSH was similar to previous reports, but lower concentrations have been reported for oxidized glutathione, methionine, and cysteine (5, 150, and 20–100 µM, respectively) [30, 32–34]. No other LMM sulfur species were evident, suggesting that if any were present in cytosol their concentrations must be < 200 µM.

### FTS included many LMM phosphorus-containing metabolites

**Fig. 7:**
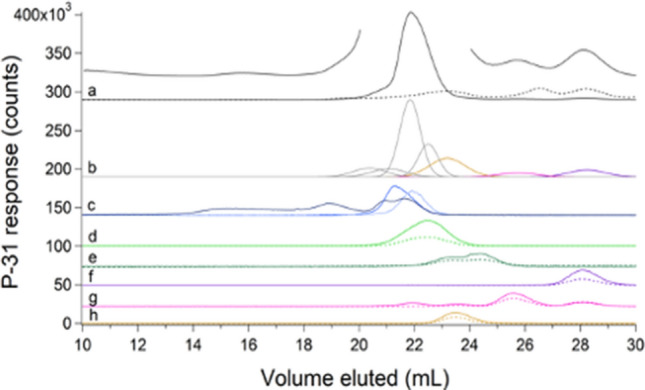
Phosphorus-detected chromatograms of FTS and standards. **a** Average traces of FTS detected by ICP-MS (solid black line ÷ 10) and at A_260_ (dashed black line × 20). The offset line is the ICP-MS data magnified × 2 excluding the dominating peak. **b** Simulations of the FTS color-coded to the standards listed below. **c** Polyphosphate after ultrafiltration (darker blue); 500 µM NaH_3_P_2_O_7_ ÷ 2 (dark blue), 500 µM Na_2_HPO_4_ × 5 (light blue); **d** 100 µM NADPH ÷ 2 (solid line) and A_260_ × 20 (dashed line); **e** 100 µM NADH × 5 (solid line) and A_260_ × 200 (dashed line); **f** 100 µM AMP × 3 (solid line) and A_260_ × 20 (dashed line); **g** 100 µM ADP and A_260_ × 20; **h** 100 µM ATP and A_260_ × 30

The FTS exhibited one intense LMM phosphorus peak (with *V*_*e*_ ≈ 22 mL) and ca. 6 minor-intensity peaks (Fig. 7, trace a). Individual traces are given in Fig. S6. Solutions of phosphorus standards Na_2_HPO_4_, NaH_3_P_2_O_7_, Na(polyphosphate) after ultrafiltration, NADPH, NADH, AMP, ADP, and ATP exhibited peaks (Fig. 7, traces c–h), some of which comigrated with those in the FTS traces (color-coded lines in Fig. 7b). Phosphate and pyrophosphate/polyphosphate traces are presented in Fig. 7c. The peaks from these standards were simulated and the same parameters were used to simulate the peaks in the FTS traces. Negative mode ESI-MS of fractions from FTS eluate established the presence of phosphates (*m*/*z* = 96.97; 194.95; 292.92), pyrophosphate (*m*/*z *= 176.94), AMP (*m*/*z* = 346.06), ADP (*m*/*z* = 426.02), ATP (*m*/*z* = 505.99) and NADH (*m*/*z* = 664.11) (Fig. S7). Inorganic phosphate ions and nucleotides were the main LMM phosphorus species in *E. coli* cytosol. The total phosphorus concentration in *E. coli* cytosolic FTS was 140 ± 40 mM. The intracellular inorganic phosphate concentration has been reported to be 1–10 mM [35]. Our concentration was probably higher because the growth media contained a high concentration of phosphate. *E. coli* cells accumulate excess phosphate ions as polyphosphate in millimolar quantities. Based on our simulations, the ATP and ADP concentrations are 1000 and 200 µM, respectively, similar to reported concentrations (1300–2000 µM and 450 µM, respectively) [32, 36]. Reported concentrations of AMP (ca. 70 µM) were dramatically lower than we observed (1000 µM). Although ESI-MS analysis established the presence of pyrophosphate, inorganic phosphates, GSSG, GSH, and other potential ligands (citrate, glutamic acid, etc.) in FTSs, no metal–ligand complexes were detected.

### Salts in FTSs suppressed ESI-MS signals

**Fig. 8:**
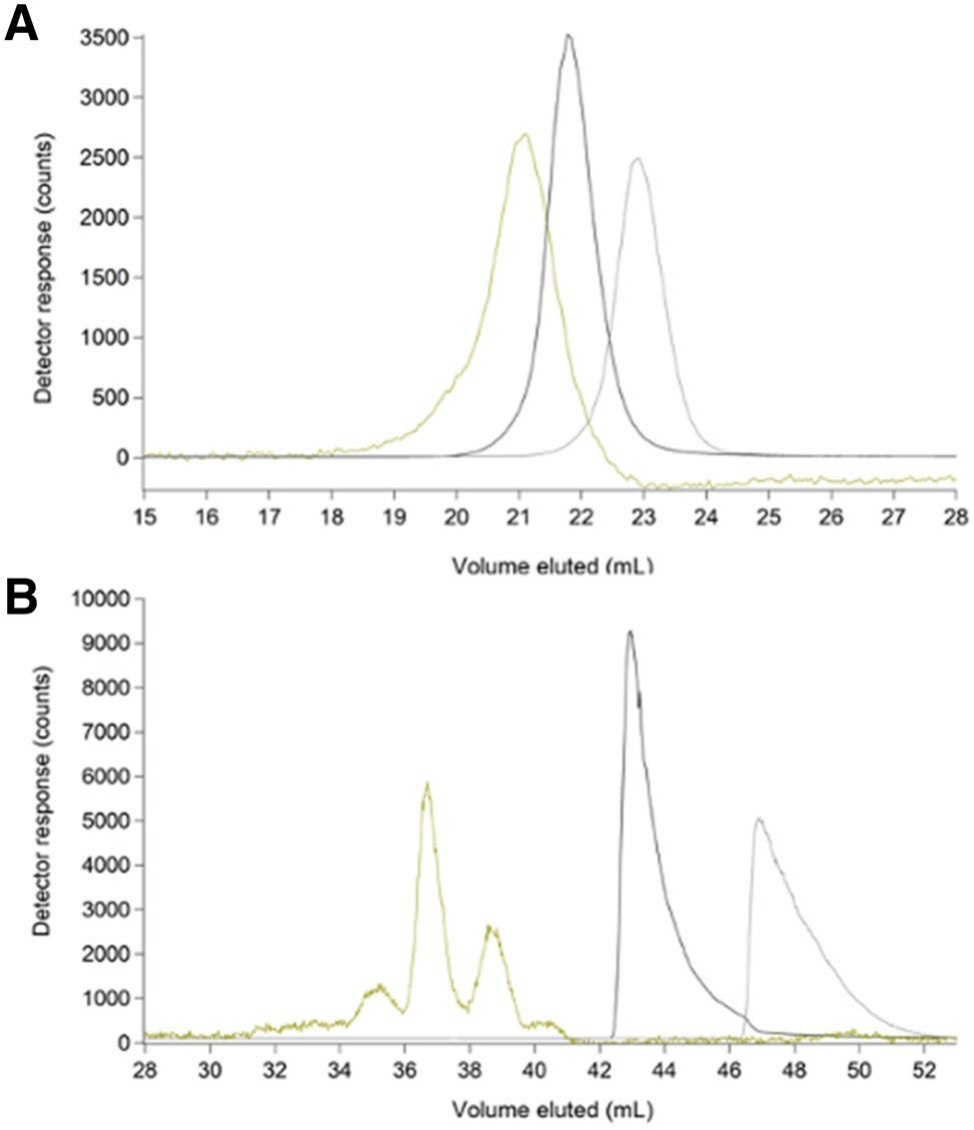
Chromatograms of FTS on a single (**a**) and double (**b**) SEC column**,** monitoring sulfur (yellow), sodium (black) ÷ 5 × 10^5^, and potassium (grey) ÷ 10^4^

We hypothesized that our inability to detect metal complexes by ESI-MS arose from the presence of salts in the FTS; “salt suppression” is a well-known mass spectrometry phenomenon [37]. To investigate, we obtained ESI-MS spectra of 0.5 mM GSH in water containing 25 mM of each salt present in the growth medium (Fig. S8). The GSH peak was uniformly observed in all samples by ESI-MS, but its intensity in salt solutions relative to that in HPW was strongly diminished. Simple inorganic salts were not well resolved from the GSH peak using the single column (Fig. 8A); however, passing *E. coli* cytosolic FTS through the double column resolved these species nicely (Fig. 8B). The improved resolution of the double column was recognized late in our study, and so the single column remained the default. Also, there was a major disadvantage of the double column, namely that processing a sample was 5 × slower than using a single column (200 min *vs*. 40 min).

### FTS consisted of 2–5 LMM iron species with a collective concentration of ~ 80 µM

Under the growth conditions used, *E. coli* cells contained 1000 ± 300 µM Fe; isolated cytosol contained 400 ± 200 µM Fe, and the cytosolic FTS contained 80 ± 20 µM Fe. Thus, the labile iron pool in these *E. coli* cells accounted for 8% of the iron in the cell and 20% of the iron in the cytosol. When the medium was supplemented with 100 µM Fe^III^ citrate, the average concentration of iron in the FTS increased to 200 µM. The range of concentrations determined in 4 independent batches was unusually large (65 µM to 500 µM) perhaps due to subtle differences in aerobicity during cell growth [21, 38]. It is also possible that some supplemented iron may not have been fully removed despite extensive washing of cells during harvesting.

**Fig. 9:**
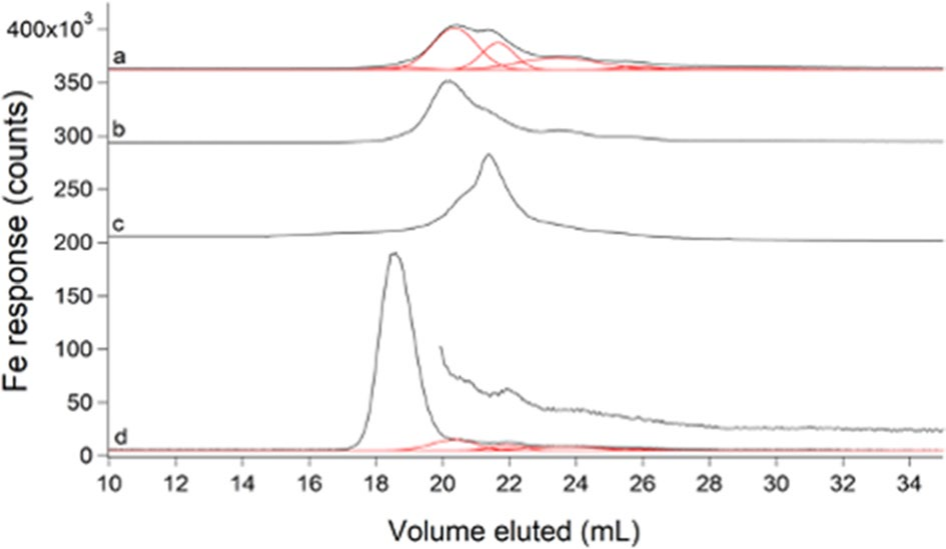
Iron-detected chromatograms of FTSs. **a** Average of 8 FTSs (black) and simulations (red); **b** average of 4 FTSs harvested during mid-exponential growth; **c** average of 4 FTS from cells supplemented with 100 µM Fe(III) citrate and harvested during mid-exponential growth; **d** un-supplemented FTS replicate incubated with 500 µM BPY and simulations for remaining peaks from **a** in red. Offset in **d** is the same trace but × 3

The average iron-detected trace of FTS revealed five partially overlapping peaks (Fig. 9, trace a). Some individual traces (Fig. S9) exhibited as few as 2 Fe species. The FTS from iron-supplemented cells exhibited similar LC peaks but with different relative intensities (Fig. 9, trace c). There was some variation in iron speciation, possibly dependent on the stage of growth during harvest. Trace b in Fig. 9 was of FTS isolated from cells harvested at mid-exponential phase. The same harvest conditions were used for supplemented growth in Fig. 9c; the shapes of the two curves were closer to each other than to the average trace. BPY was added to one FTS batch; the formation of [Fe(BPY)_3_]^2+^, and the loss of ~ 70% intensity relative to the original peaks (Fig. 9 trace d and Fig. 4A, trace b) demonstrated the lability of the original detected LMM iron-containing complexes.

We inadvertently demonstrated the lability of these complexes in another way, namely by treating FTS with acid phosphatase (PPX), which catalyzes the hydrolysis of polyphosphate chains [39]. The LMM metal species present before treatment were replaced with an increase in metals bound to PPX in the void volume (Fig. S10). This suggested that PPX chelated the LMM metal species in *E. coli* cytosol. We performed similar experiments previously to evaluate whether LMM metal species in the cytosol of *S. cerevisiae* were coordinated by polyphosphate ions [22]. At that time, we had difficulty interpreting our results (because metal polyphosphate complexes were not expected to be in the cytosol), but they can now be explained by assuming that PPX chelated metals from LMM metal complexes in the cytosol; PPX-sensitive species in the cytosol of *S. cerevisiae* probably do not coordinate polyphosphate ions.

### FTS contained 2–4 LMM copper complexes with a collective concentration of ~ 10 µM

**Fig. 10:**
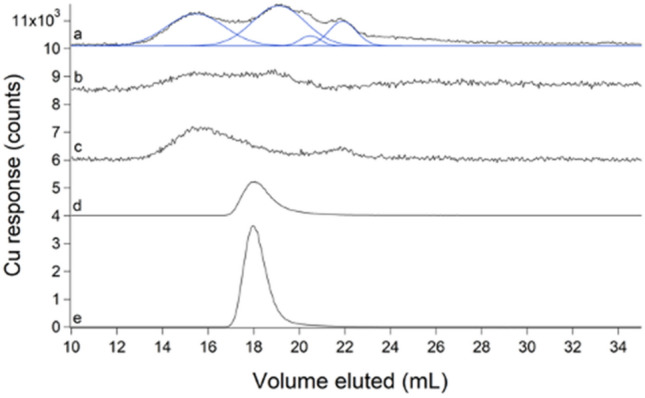
Copper-detected chromatograms of FTSs (**a–d**) and standard (**e**). **a** Average of 8 FTSs (black) with simulations in blue; **b** average of 4 FTSs from mid-exponential growth harvest; **c** FTS from cells supplemented with 1 µM of CuSO_4_ and harvested in mid-exponential phase; **d** un-supplemented FTS replicate incubated with 50 µM TPEN ÷ 100; **e** 1 µM CuSO_4_ + 10 µM TPEN ÷ 200

The average copper FTS trace consisted of 4 partially overlapping LMM species with *V*_*e*_ between 15 and 22 mL (Fig. 10, trace a). These species became more intense during late stationary phase. Some individual traces (Fig. S11) exhibited as few as 2 LMM Cu species. The modest elution volumes and relatively strong peak intensities suggested that these copper species were *not* aqueous copper ions, as such ions adsorbed strongly to the column (Fig. 2E) in contrast to the apparent undeterred passage of these species. We supplemented the growth medium with 1 µM CuSO_4_ (higher concentrations could not be used because they were toxic). Doing so increased the concentration of the LMM Cu pool from 7 ± 1 to 9.3 ± 0.2 µM Cu (comparison made for mid-exponential growth phase) and it shifted the relative intensities of the LMM peaks (Fig. 10, trace c). Again, we matched the growth phase of Cu-un-supplemented cells during harvest; the control FTS exhibited trace b in Fig. 10. Copper concentrations in whole cells and cytosol were 8 ± 2 µM and 6 ± 2 µM, respectively. The lability of the LMM copper species was established using the chelator TPEN. LC traces of TPEN-treated FTS exhibited a single peak (Fig. 10, trace d) that comigrated with a Cu(TPEN) standard (Fig. 10, trace e). We conclude, surprisingly, that *E. coli* contains a LMM *labile copper pool,* and that this pool represents the vast majority of the copper (roughly 80%!) in the cell. Some of this copper may be located in the periplasm since this is the major site of copper metabolism in *E. coli* [40].

### FTS contained LMM manganese and nickel complexes

**Fig. 11:**
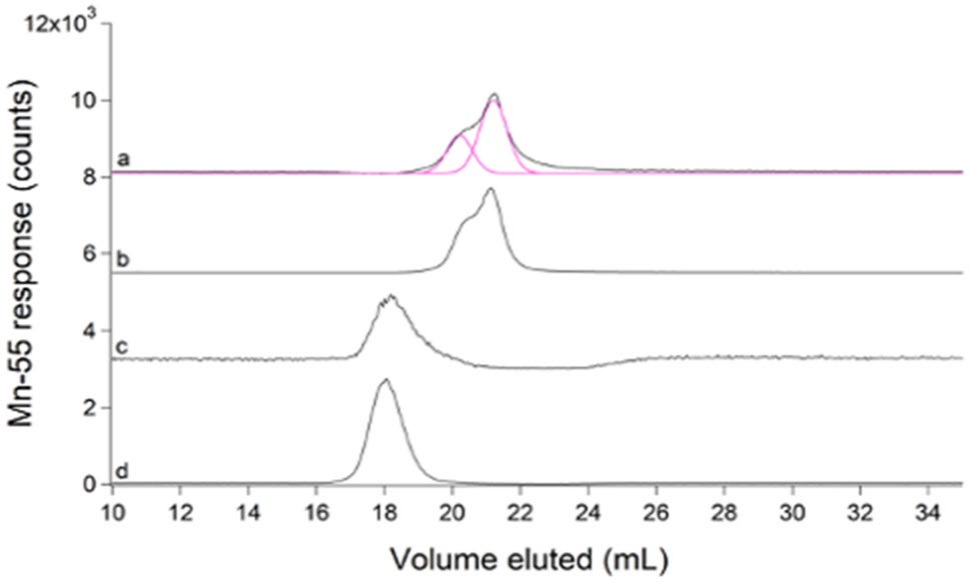
Manganese-detected chromatograms of FTSs (**a**–**c**) and standard (**d**). **a** Average of 8 FTSs (black) and simulations (pink); **b** FTS from cells supplemented with 100 µM of MnCl_2_ ÷ 100; **c** FTS incubated with 50 µM TPEN; **d** 1 µM MnCl_2_ + 10 µM TPEN ÷ 20

*Escherichia coli* FTS exhibited at least two LMM Mn peaks with *V*_*e*_ ≈ 20 and 21 mL (Fig. 11, trace a). Individual traces, shown in Fig. S12, were highly reproducible. The peak at 21 mL was about twice as intense as that at 20 mL. Under our growth conditions, *E. coli* cells and cytosol contained 7 ± 2 µM and 4 ± 1 µM Mn, respectively. The concentration of Mn in FTS was 1.4 ± 0.7 µM. Supplementing the growth medium with 100 µM MnCl_2_ in one batch increased the concentration of the labile Mn pool to 115 ± 9 µM—a 70-fold increase! The Mn trace for this batch was significantly more intense relative to un-supplemented FTSs but peak positions and relative intensities were about the same (Fig. 11, trace b). The lability of the two LMM Mn species was demonstrated using TPEN (Fig. 11c, d).

We did not focus on nickel until late in our study, but a retrospective analysis revealed two at least LMM Ni species in *E. coli* FTS, with *V*_*e*_ ≈ 19 (minor) and 20 (major) mL (Fig. S13). The concentration of Ni in FTS was 15 ± 2 µM. The corresponding sulfur trace comigrated with the Ni peaks, raising the intriguing possibility of 1–2 LMM Ni–S complex(es).

## Discussion

*Escherichia coli* and probably all/most prokaryotic and eukaryotic cells contain nonproteinaceous low-molecular-mass metal complexes that are used in metal ion trafficking, regulation, and signalling. Although the existence of these complexes has been recognized for decades, the number of species involved, their chemical composition, and their specific roles in cellular physiology remain enigmatic. The fundamental problem is that metal complexes are labile such that their ligands dissociate and reassociate rapidly. The most popular approach to study these “labile metal pools” has been and continues to be using custom-designed fluorescence-based chelator probes. Much progress has been made using chelator probes, but they destroy the complexes of interest during detection, raising doubts that such an approach can ever identify such complexes or establish their cellular roles. We are developing a complementary approach using an LC–ICP-MS system in conjunction with ESI-MS. In this study, we overcame several problems and have set the stage for future advances.

Proper sample preparation is critical for probing labile metal pools; metal chelators and buffers that coordinate metals and/or interfere with ESI-MS analysis should be excluded. EDTA is a common metal chelator that was difficult to eliminate; we did so using a strain of *E. coli* that could be lysed by a simple freeze/thaw cycle. Metals tend to interact with size-exclusion columns which contain basic sites (carboxylate groups) that bind metals. Typically, the ionic strength of the mobile phase is increased to minimize secondary column interactions, but doing so here would have been problematic for ESI-MS and ICP-MS. Thus, we invented a new strategy to combat secondary interactions, namely passing a particular isotope of aqueous zinc ions (^67^Zn) through the column which bind tightly to basic sites. Then when detecting zinc-containing eluents by ICP-MS, different zinc isotopes (^66^Zn and ^68^Zn) were monitored. Although the use of a “zinc-loaded” column did not completely inhibit all interactions and the column required periodic reloading, it minimized these problems and afforded greater reproducibility.

Using a Zn-loaded column, the chromatographic behavior of metal complexes was found to depend on the binding strength of the complex. Tight-binding complexes like [Fe(phen)_3_]^2+^ and [Fe(BPY)_3_]^2+^ passed through the column intact whereas intermediate-binding complexes like Fe(ATP) and Fe(GSH) exhibited complex behavior in which the elution profile of the metal varied with the concentration of the coordinating ligand. Weak-binding metal complexes like hexaquairon eluted slowly from the column and exhibited excessive broadening/tailing due to extensive column interactions.

We also assessed the importance of the mobile phase in chromatographic behavior; this was especially important for weak-binding metal complexes that interact strongly with the column. Mobile phase buffers with lower ionic strength promoted metal complexes to remain intact as they pass through the column but also promoted greater column interactions.

Finally, we identified salt suppression as a major problem in identifying labile metal pools by ESI-MS in aqueous cellular solutions since such solutions contain high concentrations of salts. We found that using two SEC columns linked in series was an effective, albeit time-intensive, strategy for separating LMM nonproteinaceous metal complexes from salt-containing solutions.

With these lessons learned, we assessed the labile iron, zinc, copper, manganese and nickel pools in *E. coli* cytosol as well as LMM pools of sulfur and phosphorus. As expected, the major LMM sulfur species was GSH, followed by GSSG, methionine, and cysteine. The major LMM phosphorus species in the cytosol were inorganic phosphate ions and LMM polyphosphates followed by ATP, ADP, NADH, etc. Due to the limited resolving capabilities of the single SEC column, the concentration of inorganic phosphate and LMM polyphosphates could not be determined; however, the calculated concentrations of later-eluting P species (ATP and ADP) were similar to those reported. The increased concentration of AMP observed relative to previous reports likely resulted from the DNA hydrolysis step in our cytosol isolation protocol which generates nucleotide monophosphates. While metal ions are capable of binding monophosphate groups of nucleotide monophosphates, no LMM metal species present in *E. coli* cytosol comigrated with AMP. Thus, the high concentration of nucleotide monophosphates should not have influenced the LMM metal pools.

Outten and O’Halloran concluded that WT *E. coli* cells are devoid of “free” (i.e. aqueous) Zn ions based on experiments in which Zur and ZntR transcription factors were titrated with aqueous zinc [41]. Zn-bound Zur suppresses Zn import whereas Zn-bound ZntR stimulates Zn export. An aqueous Zn concentration of 10^–15^ M minimized both activities suggesting that *E. coli* cells operate under homeostatically-regulated conditions centered around this concentration. However, this concentration corresponds to less than one atom of aqueous Zn per cell. Using our system, aqueous Zn ions interacted strongly with the column, and they eluted as broad tailing features at large volumes. In contrast, labile LMM Zn complexes with stronger ligands eluted earlier and as sharp peaks, indicating less interaction with the column. The LMM Zn peaks that we detected are in the latter category. We conclude that *E. coli* cytosol contains µM concentrations of labile LMM nonproteinaceous Zn complexes but not aqueous or “free” Zn ions. This supports Outten and O’Halloran’s conclusion that there is no “free” Zn in the cell [41], but clarifies that there is a significant *labile zinc pool* nevertheless. The LMM labile Zn species could potentially be involved in Zn trafficking and regulation, and/or perhaps metallating the Zn proteome in *E. coli*. The situation is different when *E. coli* cells are grown in media that is supplemented with Zn. In this case, the cytosol contains high concentrations (10^–5^ M) of either aqueous Zn ions or weaker binding Zn complexes that dissociate as they migrate down the column. Perhaps under Zn-stressed conditions, a secondary ligand of intermediate-binding strength sequesters excess Zn ions in the cytosol. Based on the range of Zn concentrations observed upon media supplementation, cells containing high zinc may have been harvested in a transient state in which excess Zn was being actively trafficked and subsequently exported.

*Bacillus subtilis* and other Gram-positive bacteria use bacillithiol (BSH), a sugar-based LMM molecule with cysteine and malic acid groups attached, as a Zn buffer. Zn likely coordinates to the thiol, carboxylates, and/or amide functional groups. Helmann et al. [42] have shown that the Zn(BSH) complex is strong-binding and present in the cell at sufficiently high (5 mM) concentrations such that the concentration of aqueous Zn ions should be exceedingly low. They and others have suggested that GSH plays the same buffering role in *E. coli* and in other cells that contain GSH. Besides GSH, other potential ligands for the labile zinc pool include ATP, citrate, and amino acids [6, 43], all of which have been detected by ESI-MS of fractions from FTS eluate.

Hider and Kong have presented thermodynamic-based arguments that Fe(GSH) is the dominant LMM labile Fe complex in the cytosol [30, 31]. They simulated iron complex formation in the cytosol using known affinity constants, concentrations, pH, and redox properties. In support of this, we found that solutions of iron mixed with GSH at high concentrations afforded an LC peak that eluted ca. 22 mL suggesting complex formation. However, we were unable to demonstrate that any of the detected labile iron species in FTS was Fe(GSH), perhaps due to salt suppression in our samples.

Previous studies from our lab reported two LMM iron species in *E. coli* with similar apparent masses as observed here [21]. Mössbauer spectra of the LMM iron species in *E. coli* have parameters typical of complexes with 5–6 O/N ligands—and no sulfur [21]. However, previous batches used EDTA during cytosol isolation, and we are concerned that the Fe(EDTA) complex had formed and displaced the endogenous iron complexes. Further studies are required to determine this. The concentration of LMM iron in *E. coli* was previously reported at ~ 200 µM, 2–3 times higher than observed here. However, growth conditions strongly affect the LIP concentration; for example, under aerobic conditions, the LIP concentration was only ~ 50 µM. Martin et al*.* determined a free intracellular iron concentration of 100 µM [44]. Daly et al. reported ultrafiltrate iron concentrations of just 1.2 µM [45].

Like Zn, copper trafficking in both eukaryotic and prokaryotic cells does not involve aqueous copper ions [46, 47]. Rather, copper is thought to be trafficked using copper-binding protein chaperones. Thus, the presence of nonproteinaceous LMM copper species in our FTSs was unexpected though we have recently detected similar LMM copper species in the cytosol of *S. cerevisiae* [22]. We have not chemically identified these copper species, but our results indicate that they are not simple aqueous copper ions; such ions adsorbed strongly to the column whereas the detected species eluted from the column at low *V*_*e*_ and sharp Gaussian lineshapes. Even more surprising is that the detected LMM copper species represent the majority of copper in the cell.

The labile Mn species that we detected showed little interaction with the column, suggesting strong-binding ligands, but they also migrated in the same region as aqueous Mn ions. Unstressed *E. coli* cells do not appear to use Mn, but under stressed conditions, Mn replaces iron in superoxide dismutase and ribonucleotide reductase [44]. We suggest that the detected species metallates those enzymes. WT *E. coli* grown in LB medium reportedly contain ca. 5 µM Mn (similar to what we observed), most of which was associated with MnSOD [44]. Similar to our results, Martin et al. also observed a strong increase of Mn concentration in *E. coli* (to 35 µM) when media was supplemented with Mn, and they observed whole-cell EPR indicating that this was in the Mn^II^ oxidation state. Daly et al. reported 13 µM manganese in *E. coli* cells and 0.3 µM Mn in FTSs [45]. Sharma et al. 2013 used electron spin-echo EPR and ENDOR spectroscopy to characterize the LMM Mn in *E. coli* [48]. They concluded that LMM Mn^II^ ions were coordinated by orthophosphate or other phosphorus-containing ligands, some waters, but few, if any, nitrogen-containing ligands. While comigration between Mn and P traces was observed in our cytosolic FTS, further investigation is needed to determine if the LMM Mn pool of *E. coli* is ligated by phosphorus-containing ligands.

Under anaerobic conditions, *E. coli* expresses the nickel permease system NikBCDE that imports Ni ions which are then trafficked to Ni-containing NiFe hydrogenases [49, 50]. Trafficking involves a number of metallochaperone proteins as well as a Ni(l-His)_2_ complex [51]. However, since our cells were grown aerobically, the Ni species that we detected may not be associated with these processes. Under aerobic conditions, *E. coli* needs nickel for glyoxalase, with nickel imported (inefficiently) by magnesium transporters [52, 53]. Further studies are required to establish the composition and cellular function of the detected nickel species.

In summary, the identification and characterization of labile metal pools in cells are extremely important in understanding metal ion trafficking, signalling, and regulation; however, this task is challenging due to the inherent lability of these complexes. In this study, we report major advances in overcoming problems and attaining this objective using LC–ICP-MS and ESI-MS. These advances position us closer than ever to identifying the sought-after endogenous LMM metal complexes that constitute labile metal pools in *E. coli* and other biological systems.

Major results and conclusions of this study:

- A strain of *E. coli* was employed which allowed lysates to be prepared without using EDTA.
- Pre-loading a size-exclusion chromatography column with ^67^Zn occupied sites on the column matrix that would have otherwise reacted with metal ions that migrated through the column. Then when zinc-containing samples were subsequently applied to the “zinc-loaded” column, ^66^Zn was detected and ^67^Zn, which slowly leached from the column, was ignored.
- Even using a zinc-loaded column, aqueous (or “free”) metal ions interacted with it, slowing their elution.
- A “ghost column” consisting of PEEK tubing that replaced the actual column was used to evaluate the proportion of metal ions in a sample that adhered to the column. The degree of column interaction varied approximately with the Irving–Williams series: Mn (least) < Fe < Ni < Zn < Cu (most)
- The mobile-phase buffer impacted chromatographic behavior; lower ionic strength promoted metal complexes to remain intact as they pass through the column but also promoted greater column interactions.
- The chromatographic behavior of iron and zinc standards reflected the M–L binding strength of the complex; thermodynamically stable complexes (e.g. bound to 1,10-phenanthroline and 2,2′-bipyridyl) held together as they migrated down the column whereas less stable complexes [e.g. Fe^II^(ATP) and Fe^II^(GSH)] showed evidence of some dissociation (shifting elution volumes with changing ligand:metal ratios and mobile phase buffer composition).
- With low-molecular-mass standards (less than ~ 1000 Da), elution volumes did not follow calibration curves, such that all masses obtained using such curves should be considered *apparent*.
- The Zn(GSH) complex migrated in-tact through the column, but only at high GSH concentrations. This complex was detected by ESI-MS.
- The LMM sulfur pool in cytosol consisted of GSH, GSSG, methionine and cysteine, as confirmed by ESI-MS. Approximate cytosolic concentrations were 3000, 400, 800, and 200 µM, respectively.
- The LMM phosphorus pool in cytosol consisted of 1 intense LC peak and ca. 6 minor peaks. ESI-MS established the presence of phosphates, pyrophosphate, AMP, ADP, ATP and NADH. The dominant LC peak probably arose from phosphate ions and nucleotides. Estimated ATP, ADP, and AMP concentrations were 1000, 200, and 1000 µM, respectively.
- No endogenous Fe, Zn, Cu, or Mn complexes in cytosol were detected by ESI-MS, probably due to salt suppression and low concentrations. Salt suppression was a major problem because cytosol and other cell compartments contain high concentrations of salts. Using a longer column diminished this problem but also diluted samples.
- Cytosolic flow-through-solution from *E. coli* cells contained 2–5 labile LMM zinc complexes with a collective concentration of ~ 13 µM. Supplementing the growth medium with 100 µM Zn acetate led to a Zn pool concentration of ~ 200 µM.
- The *E. coli* cytosol consists of 2–5 LMM iron species with a collective concentration of ~ 80 µM. For the growth conditions used, this represented 8% of the iron in the cell and 20% of the iron in the cytosol. When cells were grown on high iron, the concentration of iron in the FTS increased to ~ 200 µM.
- After the FTS was treated with BPY, iron LC peaks declined ~ 70% indicating that the detected peaks were labile as commonly defined.
- FTS contained 2–4 LMM copper complexes with a collective concentration of ~ 10 µM. These complexes are not aqueous (or “free”) copper; such ions adhere tightly to the column. Increasing the copper concentration in the growth medium had little effect on the copper concentration in the FTS, but the relative intensities of the peaks changed. Surprisingly, *E. coli* contains a LMM labile copper pool that represents the vast majority (~ 80%) of the copper in the cell.
- FTS contained 2 LMM manganese species with a collective concentration of ~ 1.4 µM. These species represented ~ 20% of the manganese in the cell. Supplementing Mn in the growth increased the Mn concentration in the FTS hugely.
- FTS contained ~ 2 LMM Ni species with a collective concentration of ~ 15 µM. These peaks comigrated with sulfur suggesting LMM Ni–S complex(es) in *E. coli*.

## Supplementary Information

Below is the link to the electronic supplementary material.

Supplementary file1 (PDF 919 kb)

## Abbreviations

AA: Ammonium acetate
BPY: 2,2-Bipyridine
CV: Column volume
EDTA: Ethylenediaminetetraacetic acid
EGTA: Ethylene glycol-bis(β-aminoethyl ether)-*N*,*N*,*N*′,*N*′-tetraacetic acid
ESI-MS: Electrospray ionization mass spectrometry
FTS: Flow-through solution
GSH: Glutathione
GSSG: Oxidized glutathione
HPW: High-purity water
ICP-MS: Inductively coupled plasma mass spectrometry
LC: Liquid chromatography
LIP: Labile iron pool
LMM: Low-molecular-mass
phen: 1,10-Phenanthroline
SEC: Size-exclusion chromatography
TPEN: *N*,*N*,*N*′,*N*′-Tetrakis(2-pyridinylmethyl)-1,2-ethanediamine

## Declarations

### Availability of data and material

Chromatography data are available upon request.

## References

[1] Finney LA, O’Halloran TV (2003). Transition metal speciation in the cell; insights from the chemistry of metal ion receptors. Science.

[2] Imlay JA (2003). Pathways of oxidative damage. Ann Rev Microbiol.

[3] Macomber L, Imlay JA (2009). The iron-sulfur clusters of dehydratases are primary intracellular targets of copper toxicity. Proc Natl Acad Sci USA.

[4] Imlay JA (2014). The mismetallation of enzymes during oxidative stress. J Biol Chem.

[5] Gu M, Imlay JA (2013). Superoxide poisons mononuclear iron enzymes by causing mismetallation. Mol Microbiol.

[6] Krężel A, Maret W (2016). The biological inorganic chemistry of zinc ions. Arch Biochem Biophys.

[7] Lindahl PA, Moore MJ (2016). Labile low-molecular-mass metal complexes in mitochondria: trials and tribulations of a burgeoning field. Biochemistry.

[8] O’Halloran TV, Culotta VC (2000). Metallochaperones, an intracellular shuttle service for metal ions. J Biol Chem.

[9] Ma Z, Jacobsen FE, Giedroc DP (2009). Coordination chemistry of bacterial metal transport and sensing. Chem Rev.

[10] Wilson S, Bird AJ (2016). Zinc sensing and regulation in yeast model systems. Arch Biochem Biophys.

[11] Jacobs A (1977). Low-molecular weight intracellular iron transport compounds. Blood.

[12] Williams RJP (1982). Free manganese(II) and iron(II) cations can act as intracellular cell controls. FEBS Lett.

[13] Crichton (RR),  (1984). Iron uptake and utilization by mammalian cells II. Intracellular iron utilization. Trends Biochem Sci.

[14] Lisher JP, Giedroc DP (2013). Manganese acquisition and homeostasis at the host-pathogen interface. Front Cell Infect Microbiol.

[15] Braymer JJ, Giedroc DP (2014). Recent developments in copper and zinc homeostasis in bacterial pathogens. Curr Opin Chem Biol.

[16] Petrat F, de Groot H, Sustmann R, Rauen U (2002). The chelatable iron pool in living cells: a methodically defined quantity. Biol Chem.

[17] Petrat F, Rauen U, de Groot H (1999). Determination of the chelatable iron pool of isolated rat hepatocytes by digital fluorescence microscopy using the fluorescent probe, phen green SK. Hepatology.

[18] Zastrow ML, Huang Z, Lippard SJ (2020). HaloTag-Based Hybrid Targetable and Ratiometric Sensors for Intracellular Zinc. ACS Chem Biol.

[19] Chung CYS, Posimo JM, Lee S, Tsang T, Davis JM, Brady DC, Chang CJ (2019). Activity-based ratiometric FRET probe reveals oncogene-driven changes in labile copper pools induced by altered glutathione metabolism. Proc Natl Acad Sci USA.

[20] Carter KP, Young AM, Palmer AE (2014). Fluorescent sensors for measuring metal ions in living systems. Chem Rev.

[21] Wofford JD, Bolaji N, Dziuba N, Outten FW, Lindahl PA (2019). Evidence that a respiratory shield in *Escherichia coli* protects a low molecular mass Fe^II^ pool from O_2_-dependent oxidation. J Biol Chem.

[22] Nguyen TQ, Kim JE, Brawley HN, Lindahl PA (2020). Chromatographic detection of low-molecular-mass metal complexes in the cytosol of *Saccharomyces cerevisiae*. Metallomics.

[23] Dziuba N, Hardy J, Lindahl PA (2019). Low-molecular-mass iron complexes in blood plasma of iron-deficient pigs do not originate directly from nutrient iron. Metallomics.

[24] McCormick SP, Moore MJ, Lindahl PA (2015). Detection of labile low-molecular-mass transition metal complexes in mitochondria. Biochemistry.

[25] Cahill J, Young R (2019). Phage lysis: multiple genes for multiple barriers. Adv Virus Res.

[26] Hellberg U, Ivarsson JP, Johansson BL (1996). Characteristics of Superdex® prep grade media for gel filtration chromatography of proteins and peptides. Process Biochem.

[27] Irving H, Williams RJP (1948). Order of stability of metal complexes. Nature.

[28] Weaver J, Pollack S (1989). Low-Mr iron isolated from guinea pig reticulocytes as AMP-Fe and ATP-Fe complexes. Biochem J.

[29] Smith RM, Martell AE (1990). Critical stability constants.

[30] Hider RC, Kong XL (2011). Glutathione: a key component of the cytoplasmic labile iron pool. Biometals.

[31] Hider RC, Kong XL (2013). Iron speciation in the cytosol: an overview. Dalton Trans.

[32] Bennett BD, Kimball EH, Gao M, Osterhout R, Van Dien SJ, Rabinowitz JD (2009). Absolute metabolite concentrations and implied enzyme active site occupancy in *Escherichia coli*. Nature Chem Biol.

[33] Helbig K, Bleuel C, Krauss GJ, Nies DH (2008). Glutathione and transition-metal homeostasis in *Escherichia Coli*. J Bacteriol.

[34] Smirnova GV, Tyulenev AV, Bezmaternyk KV, Muzyka NG, Ushakov VY, Oktyabrsky ON (2019). Cysteine homeostasis under inhibition of protein synthesis in *Escherichia coli* cells. Amino Acids.

[35] McCleary WR (2017). Molecular mechanisms of phosphate homeostasis in *Escherichia coli*. Recent Adv Physiol Pathog Biotechnol Appl.

[36] Zbornickova E, Knejzlika R, Hauryliuk V, Krasny L, Rejman D (2019). Analysis of nucleotide pools in bacteria using HPLC-MS in HILIC mode. Talanta.

[37] Piwowar AM, Lockyer NP, Vickerman JC (2009). Salt effects on ion formation in desorption mass spectrometry: an investigation into the role of alkali chlorides on peak suppression in Time-of-Flight-Secondary Ion Mass Spectrometry. Anal Chem.

[38] Beauchene NA, Mettert EL, Moore LJ, Keles S, Willey ER, Kiley PJ (2017). O_2_ availability impacts iron homeostasis in *Escherichia coli*. Proc Natl Acad Sci USA.

[39] Akiyama M, Crooke E, Kornberg A (1993). An exopolyphosphatase of *Escherichia coli*. the enzyme and its ppx gene in a polyphosphate operon. J Biol Chem.

[40] Rensing C, Grass G (2003). *Escherichia coli* mechanisms of copper homeostasis in a changing environment. FEMS Microbiol Rev.

[41] Outten CE, O’Halloran TV (2001). Femtomolar sensitivity of metalloregulatory proteins controlling zinc homeostasis. Science.

[42] Ma P, Chandrangsu TC, Helmann A, Romsang A, Gaballa A, Helmann JD (2014). Bacillithiol is a major buffer of the labile zinc pool in *Bacillus subtilis*. Mol Microbiol.

[43] Glover CN, Bury NR, Hogstrand C (2003). Zinc uptake across the apical membrane of freshwater rainbow trout intestine is mediated by high affinity, low affinity, and histidine-facilitated pathways. Biochim Biophys ACTA.

[44] Martin JE, Waters LS, Storz G, Imlay JA (2015). The *Escherichia coli* Small protein MntS and exporter MntP optimize the intracellular concentration of manganese. PLoS Genet.

[45] Daly MJ, Gaidamakova EK, Matrosova VY, Kiang JG, Fukumoto R, Lee DY, Wehr NB, Viteri GA, Berlett BS, Levine RL (2010). Small-molecule antioxidant proteome-shields in *Deinococcus radiodurans*. PLoS ONE.

[46] Rae TD, Schmidt PJ, Pufahl RA, Culotta VC, O’Halloran TV (1999). Undetectable intracellular free copper: The requirement of a copper chaperone for superoxide dismutase. Science.

[47] Changela A, Chen K, Xue Y, Holschen J, Outten CE, O'Halloran TV, Mondragón A (2003). Molecular basis of metal-ion selectivity and zeptomolar sensitivity by CueR. Science.

[48] Sharma A, Gaidamakova EK, Matrosova VY, Bennett B, Daly MJ, Hoffman BM (2013). Responses of Mn^2+^ speciation in *Deinoccoccus radiodurans* and *Escherichia coli* to γ-radiation by advanced paramagnetic resonance methods. Proc Natl Acad Sci USA.

[49] Higgins KA, Carr CE, Maroney MJ (2012). Specific metal recognition in nickel trafficking. Biochemistry.

[50] Miki K, Atomi H, Watanabe S (2020). Structural Insight into [NiFe] hydrogenase maturation by transient complexes between Hyp Proteins. Acc Chem Res.

[51] Chivers PT, Benanti EL, Heil-Chapdelaine V, Iwig JS, Rowe JL (2012). Identification of Ni-(L-His)(2) as a substrate for NikABCDE-dependent nickel uptake in *Escherichia coli*. Metallomics.

[52] Boer JL, Mulrooney SB, Hausinger RP (2014). Nickel-dependent metalloenzymes. Arch Biochem Biophys.

[53] Moncrief MB, Maguire ME (1999). Magnesium transport in prokaryotes. J Biol Inorg Chem.

