# Supplementary material for: Low-molecular-mass labile metal pools in Escherichia coli: advances using chromatography and mass spectrometry
Source: J Biol Inorg Chem. 2021 May 8;26(4):479–94. doi: 10.1007/s00775-021-01864-w (PMC8205893; doi:10.1007/s00775-021-01864-w)

Supplementary Information

Title: **Low-Molecular-Mass Labile Metal Pools in *Escherichia Coli*: Advances using Chromatography and Mass Spectrometry**

Authors: Hayley N. Brawley and Paul A. Lindahl

Table of Contents:

1. Table S1. Standards for molecular mass calibration curve using single column.

2. Table S2. Parameters used to simulate chromatography peaks.

3. Figure S1. Zinc-detected chromatographic traces of individual batches of FTS.

4. Figure S2: Phosphorus-detected chromatographic traces of $Na_2ATP$ using 50 mM AA or 20 mM AA as the mobile phase.

5. Figure S3. Zinc- and Sulfur-detected traces showing no Zn(GSH) complex in cytosolic FTS.

6. Figure S4. Sulfur-detected chromatographic traces of individual batches of FTS.

7. Figure S5. Positive mode ESI-MS spectra showing sulfur-containing metabolites in cytosolic FTS.

8. Figure S6. Phosphorus-detected chromatographic traces of individual batches of FTS.

9. Figure S7. Negative mode ESI-MS spectra showing phosphorus-containing metabolites in cytosolic FTS.

10. Figure S8. Positive mode ESI-MS spectra showing salt suppression of GSH.

11. Figure S9. Iron-detected chromatographic traces of individual batches of FTS.

12. Figure S10. Acid phosphatase chelates metals from LMM metal complexes.

13. Figure S11. Copper-detected chromatographic traces of individual batches of FTS.

14. Figure S12. Manganese-detected chromatographic traces of individual batches of FTS.

15. Figure S13. Averaged FTS traces of Ni, S, and P.

**Table S1. Standards for molecular mass calibration curve using single column.**

| Compound | Source | Method of detection | Molecular weight (Da) | Concentration | Log(MW) | Elution volume (mL) | $V_e/V_o$ |
|---|---|---|---|---|---|---|---|
| $(Na)_2$AMP | see *Metal and ligand standards* | P, UV-260 | 347 | 100 µM | 2.54 | 28.53 | 1.37 |
| Riboflavin | Sigma Aldrich | UV-260 | 376 | 1 mM | 2.58 | 26.59 | 2.65 |
| NaADP | see *Metal and ligand standards* | P, UV-260 | 427 | 100 µM | 2.63 | 25.87 | 1.55 |
| $(Na)_2$ATP | see *Metal and ligand standards* | P, UV-260 | 507 | 100 µM | 2.71 | 23.89 | 2.07 |
| $(Na)_2$NADH | see *Metal and ligand standards* | P, UV-260 | 663 | 50 µM | 2.82 | 24.67 | 2.44 |
| $(Na)_4$NADPH | see *Metal and ligand standards* | P, UV-260 | 744 | 50 µM | 2.87 | 22.74 | 2.57 |
| Cyanocobalamin | Fisher Bioreagents | Co | 1,355 | 50 µM | 3.13 | 19.26 | 2.78 |
| Insulin (bovine) | Sigma Aldrich | UV-280 | 5,734 | 50 µM | 3.76 | 14.46 | 3.06 |
| Cytochrome C (equine) | Sigma Aldrich | Fe | 12,384 | 20µM | 4.09 | 12.74 | 2.86 |
| Thyroglobulin | Sigma Aldrich | UV-210 | 660,000 | 0.1 mg/ml | 5.82 | 9.31 | 1.00 |
| $NaH_2PO_4$ | see *Metal and ligand standards* | Fe | 95 | 20 µM | 1.98 | 21.92 | 2.35 |
| Cysteine | see *Metal and ligand standards* | S | 121 | 500 µM | 2.08 | 22.15 | 2.38 |
| Methionine | see *Metal and ligand standards* | S | 149 | 500 µM | 2.17 | 21.19 | 2.28 |
| GSH | see *Metal and ligand standards* | S | 307 | 100 µM | 2.49 | 21.09 | 2.27 |
| ZnEDTA | Acros Organics/Sigma Aldrich | Zn | 354 | 1/10 µM | 2.55 | 19.74 | 2.12 |
| Fe(Bipy)3 | Fisher Chemical/Alfa Aesar | Fe, UV-523 | 524 | 2/20 µM | 2.72 | 18.44 | 1.98 |
| Fe(Phen)3 | Fisher Chemical/Acros Organics | Fe, UV-510 | 597 | 2/20 µM | 2.78 | 21.19 | 2.28 |
| GSSG | see *Metal and ligand standards* | S | 613 | 100 µM | 2.79 | 20.03 | 2.15 |
| Zn acetate (hexaaqua Zn) | see *Metal and ligand standards* | Zn | 172 | 1 µM | 2.24 | 44.89 | 4.82 |
| $FeSO_4$ (hexaaqua Fe) | see *Metal and ligand standards* | Fe | 164 | 1 µM | 2.21 | 22.40 | 2.41 |
| $MnCl_2$ (hexaaqua Mn) | see *Metal and ligand standards* | Mn | 163 | 1 µM | 2.21 | 20.71 | 2.22 |
| $NiSO_4$ (hexaaqua Ni) | see *Metal and ligand standards* | Ni | 166 | 1 µM | 2.22 | 24.98 | 2.68 |

**Table S2. Parameters used to simulate chromatography peaks.** FWHM, full width at half maximum. The areas listed for simulations of Figures 1Ad and 9D do not sum to 100% because the dominant peaks in those traces were no

| Zn (figure 1) | | |
|---|---|---|
| Center (mL) | Area (%) | FWHM (mL) |
| (Ac) 20.3 | 26 | 1.4 |
| (Ac) 21.9 | 15 | 1.0 |
| (Ac) 24.1 | 30 | 3.3 |
| (Ac) 26.1 | 4 | 1.4 |
| (Ac) 31.3 | 25 | 4.5 |
| (Ad) 20.2 | 2 | 1.8 |
| (Ad) 23.9 | 1 | 3.3 |
| (Ad) 25.7 | 0.4 | 2.0 |
| (Ad) 30.5 | 2 | 4.5 |
| Fe (figure 5) | | |
| (Ab) 23.3 | 23 | 1.3 |
| (Ac) 22.3 | 38 | 1.2 |
| (Ac) 23.4 | 35 | 1.4 |
| (Bb) 28.0 | 12 | 1.4 |
| (Bc) 26.6 | 53 | 1.4 |
| (Bc) 28.0 | 32 | 1.4 |
| S (figure 6) | | |
| Center (mL) | Area (%) | FWHM (mL) |
| 20.1 | 28 | 1.6 |
| 21.1 | 51 | 1.2 |
| 21.3 | 14 | 0.8 |
| 22.1 | 7 | 1.4 |
| P (figure 7) | | |
| Center (mL) | Area (%) | FWHM (mL) |
| 20.4 | 2 | 1.4 |
| 21.1 | 9 | 1.4 |
| 21.8 | 57 | 0.9 |
| 22.5 | 24 | 0.8 |
| 23.2 | 5 | 1.5 |
| 25.8 | 1 | 1.6 |
| 28.2 | 2 | 1.5 |
| Fe (figure 9) | | |
| Center (mL) | Area (%) | FWHM (mL) |
| (A) 20.4 | 51 | 2.0 |
| (A) 21.8 | 18 | 1.2 |
| (A) 23.6 | 24 | 3.3 |
| (A) 26.1 | 2 | 1.4 |
| (A) 28.3 | 5 | 4.6 |
| (D) 20.4 | 6 | 1.6 |
| (D) 21.9 | 3 | 1.1 |
| (D) 23.6 | 4 | 2.8 |
| (D) 25.9 | 1 | 1.4 |
| Cu (figure 10) | | |
| Center (mL) | Area (%) | FWHM (mL) |
| 15.4 | 38 | 3.0 |
| 19.1 | 44 | 2.7 |
| 20.5 | 4 | 1.0 |
| 21.9 | 14 | 1.4 |
| Mn (figure 11) | | |
| Center (mL) | Area (%) | FWHM (mL) |
| 20.2 | 32 | 0.9 |
| 21.2 | 68 | 0.9 |

**Figure S1. Zinc-detected chromatographic traces of individual batches of FTS.** Replicates are listed in order from highest $OD_{600}$ (top) at which cells were harvested to lowest (bottom). Blue horizontal lines separate the different exponential growth phases. Dashed vertical lines highlight LMM Zn species with $V_e/V_o$. R1-R10 are individual batches numbered chronologically.

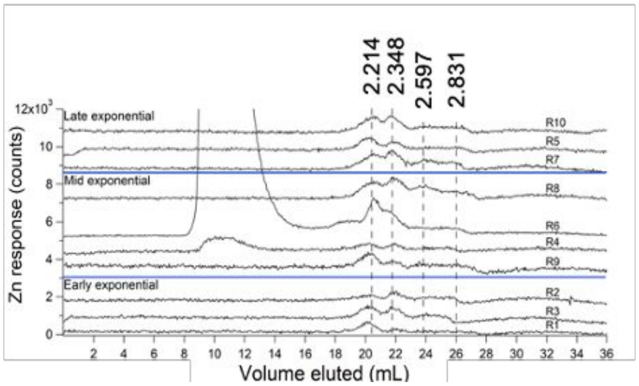

**Figure S2: Phosphorus-detected chromatographic traces of $Na_2ATP$ using 50 mM AA (A) or 20 mM AA (B) as the mobile phase.** In A and B, the solid-line traces were from solutions containing 1 µM $FeSO_4$ + variable (final) concentrations of $Na_2ATP$, as follows in units of µM ATP. Panel A and B: (a), 5 ×200; (b), 10 ×100; (c), 25 ×40; (d), 50 ×20; (f), 500 ×2; (g), 1000. In A and B, the dashed-line traces were from solutions of $Na_2ATP$ without added iron at the following concentrations (µM): Panel A and B: (a), 5 ×200; (b), 10 ×100; (c), 25 ×40; (d), 50 ×20; (e), 100 ×10; (f), 500 ×2; (g), 1000.

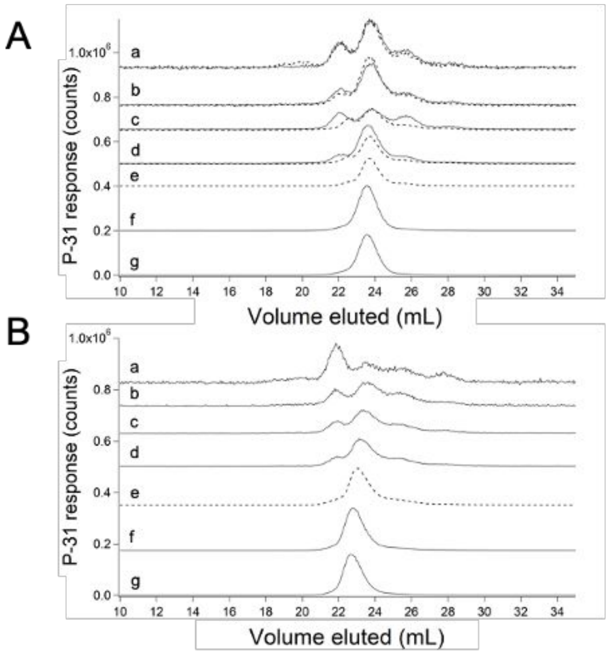

**Figure S3. Zinc- and Sulfur-detected traces showing no Zn(GSH) complex in cytosolic FTS.** (A), averaged FTS from cells supplemented with 100 μM Zn acetate (green line, Zn detected; blue line, S detected); (B), 100 μM GSH (blue) ×3 and 2 μM Zn acetate (green); (C), 2 μM Zn acetate (green) + 1 mM GSH (blue); (D), 2 μM Zn acetate (green) + 100 mM GSH (blue ÷100). Traces (B) – (D) were standards prepared in mobile phase buffer.

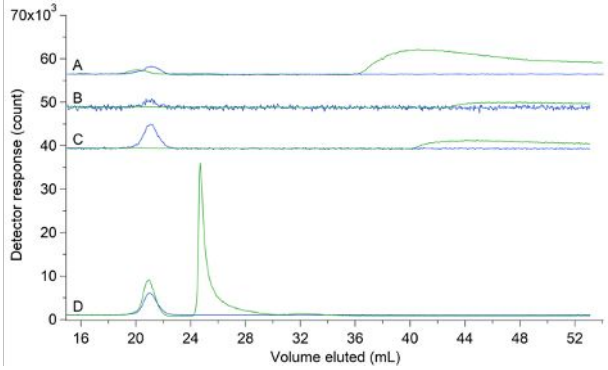

**Figure S4. Sulfur-detected chromatographic traces of individual batches of FTS.** Replicates in order from highest $OD_{600}$ (top) at which cells were harvested to lowest (bottom). Blue horizontal lines separate the different exponential growth phases. Dashed vertical lines highlight LMM S species with $V_e/V_o$.

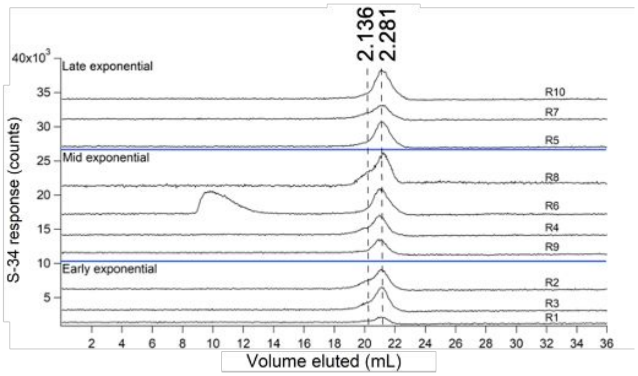

**Figure S5. Positive mode ESI-MS spectra showing Sulfur-containing metabolites in cytosolic FTS.** (A), Sulfur-detected averaged FTS from cells collected on the double peptide column (dashed lines indicated fraction boundaries). (B), Positive mode ESI of selected fractions from (A). Peaks within each column have been normalized to the largest peak intensity.

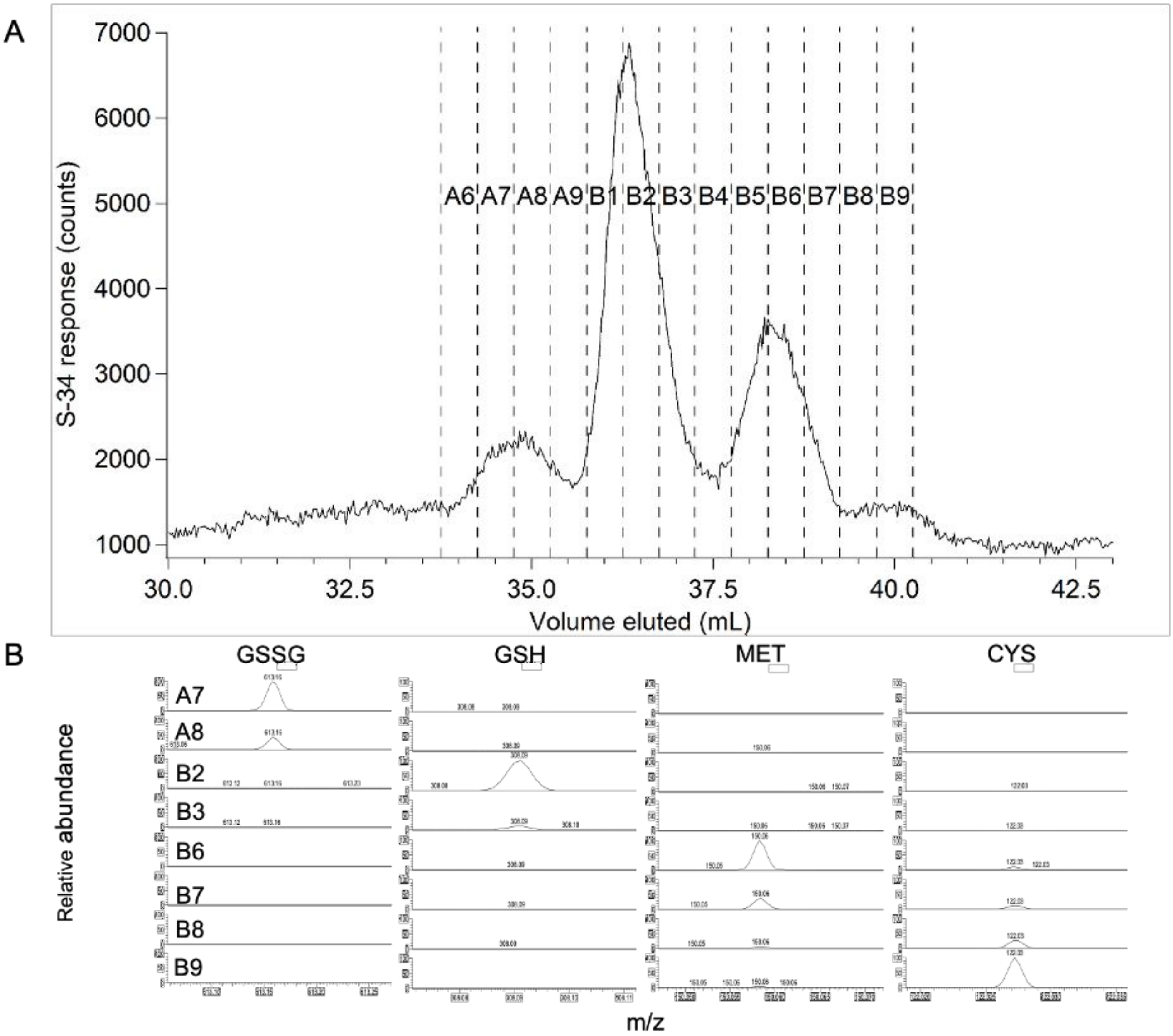

**Figure S6. Phosphorus-detected chromatographic traces of individual batches of FTS.** Panel A: replicates in order from highest $OD_{600}$ (top) at which cells were harvested to lowest (bottom). Panel B: Same replicates as in Panel A with main peak removed. Blue horizontal lines separate the different exponential growth phases. Dashed vertical lines highlight LMM P species with $V_e/V_o$.

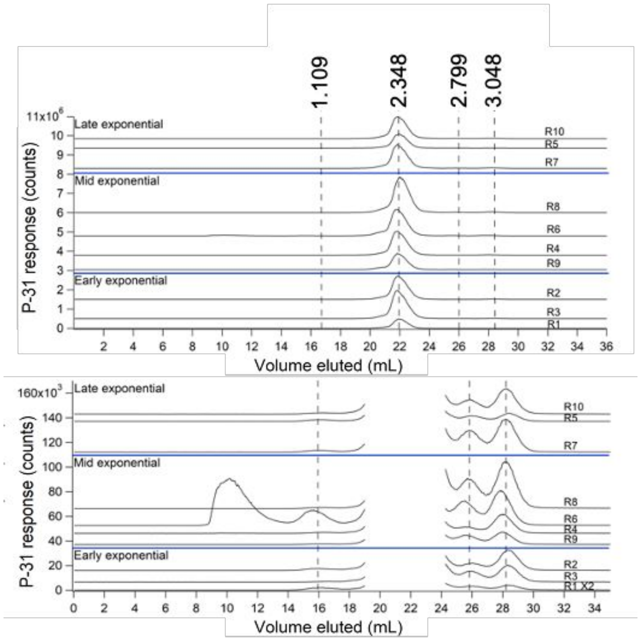

**Figure S7. Negative mode ESI-MS spectra showing Phosphorus-containing metabolites in cytosolic FTS.** (A), Phosphorus-detected averaged FTS from cells collected on single peptide (dashed lines indicated fraction boundaries). The offset line is the ICP-MS data magnified ×20 excluding the dominating peak. (B), Negative mode ESI of selected fractions from (A). Peaks within each column have been normalized to the largest peak intensity.

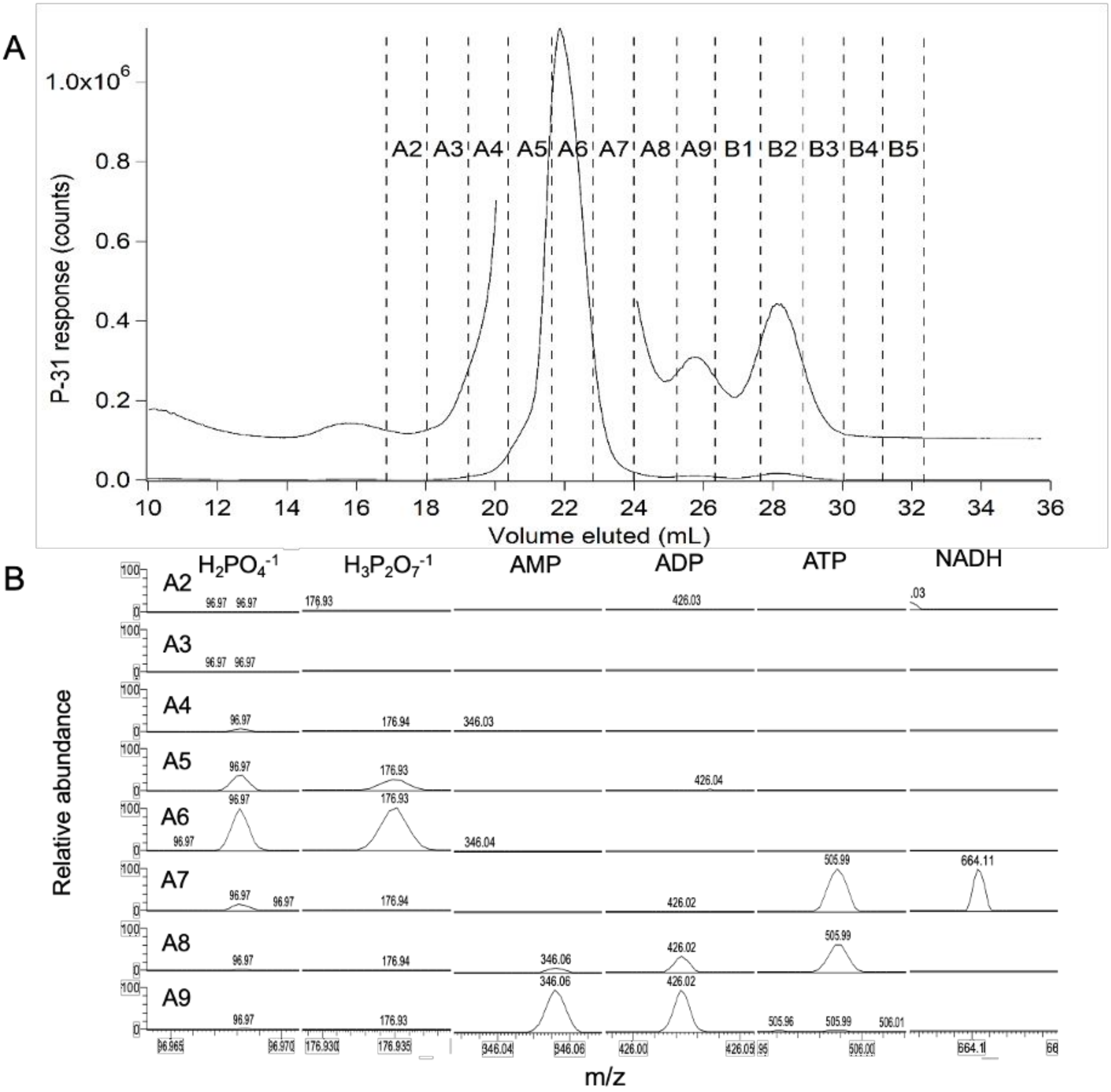

**Figure S8. Positive mode ESI-MS spectra showing salt suppression of GSH.** (A), 500 µM GSH; (B), a FTS replicate 8 (R8) (C), the sulfur-containing fraction collected from the FTS replicate in (B); (D), 500 µM GSH + 25 mM $Na_2HPO_4$; (E), 500 µM GSH + 25 mM $KH_2PO_4$; (F), 500 µM GSH + 25 mM NaCl; (G), 500 µM GSH + 25 mM $MgSO_4$. Spectra were normalized to the peak intensity of the GSH standard in (A). Relative percentage intensities for (B) – (G) are indicated.

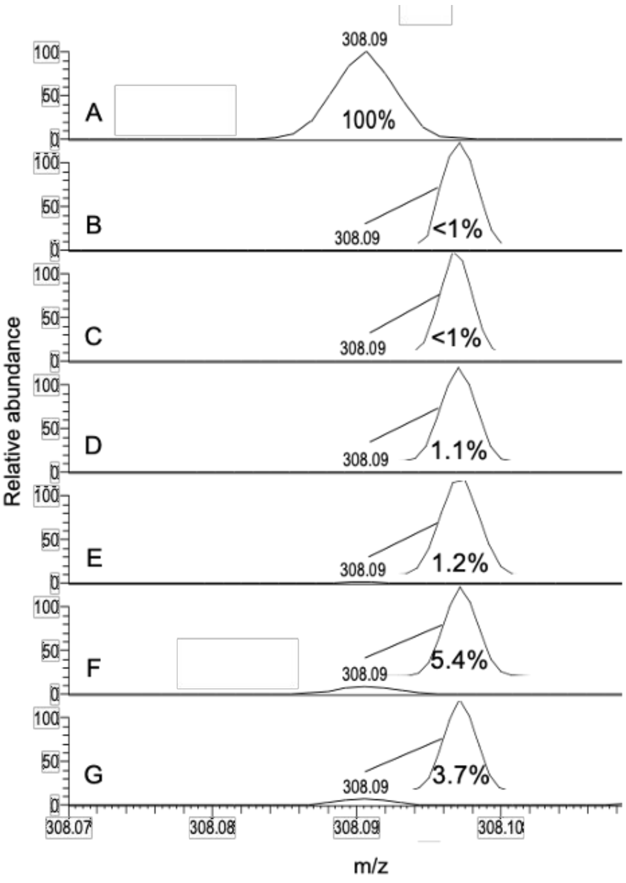

**Figure S9. Iron-detected chromatographic traces of individual batches of FTS.** Replicates in order from highest $OD_{600}$ (top) at which cells were harvested to lowest (bottom). Blue horizontal lines separate the different exponential growth phases. Dashed vertical lines highlight LMM Fe species with $V_e/V_o$.

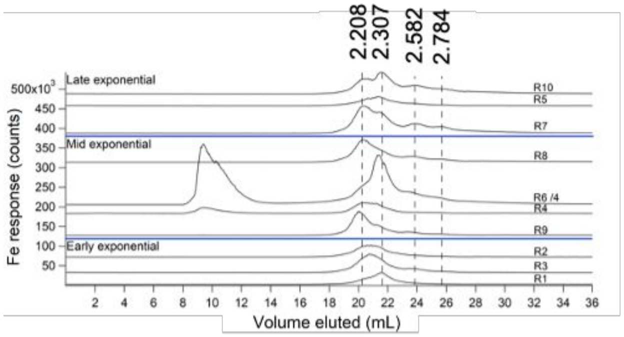

**Figure S10. Acid phosphatase chelates metals from LMM metal complexes.** Solid, dark-colored traces are the average of 8-10 FTSs for Fe (red, ÷4), Zn (green), Cu (blue), Mn (pink), and P (black, ÷100). Solid, light-colored traces are FTS + 2 mg/mL acid phosphatase for (same scaling for average FTSs). Dashed traces are metal content of standard 2 mg/mL acid phosphatase.

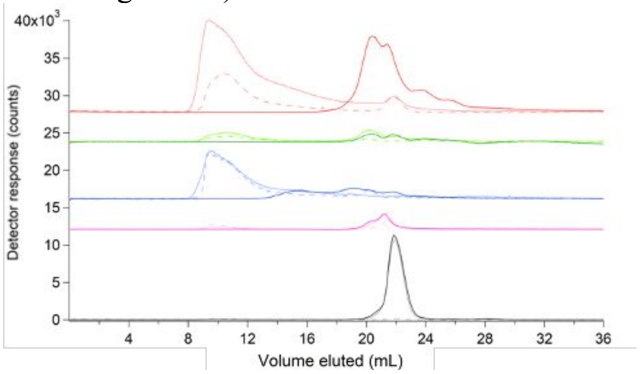

**Figure S11. Copper-detected chromatographic traces of individual batches of FTS (R1 – R10).** Replicates in order from highest $OD_{600}$ (top) at which cells were harvested to lowest (bottom). Blue horizontal lines separate the different exponential growth phases. Dashed vertical lines highlight LMM Cu species with $V_e/V_o$.

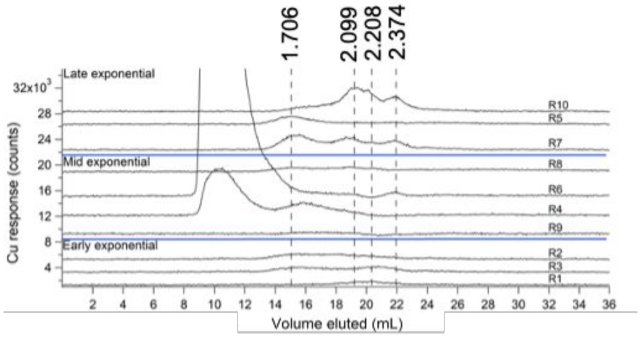

**Figure S12. Manganese-detected chromatographic traces of individual batches of FTS (R1 – R10).** Replicates in order from highest $OD_{600}$ (top) at which cells were harvested to lowest (bottom). Blue horizontal lines separate the different exponential growth phases. Dashed vertical lines highlight LMM Mn species with $V_e/V_o$.

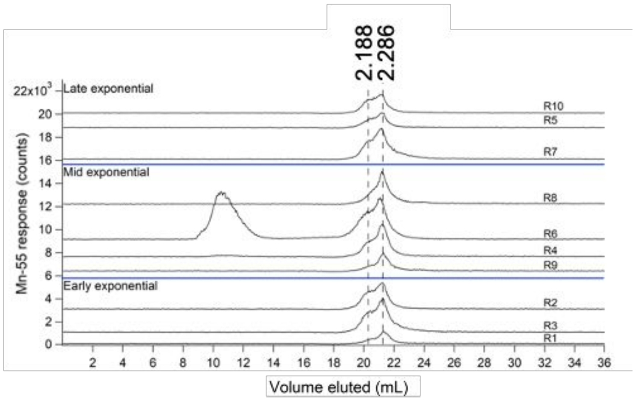

**Figure S13. Average of 9 – 10 FTS traces of Ni (purple), S (yellow), and P (black)** ÷100. The offset line in the bottom trace has been magnified ×10 with the dominating peak excluded.

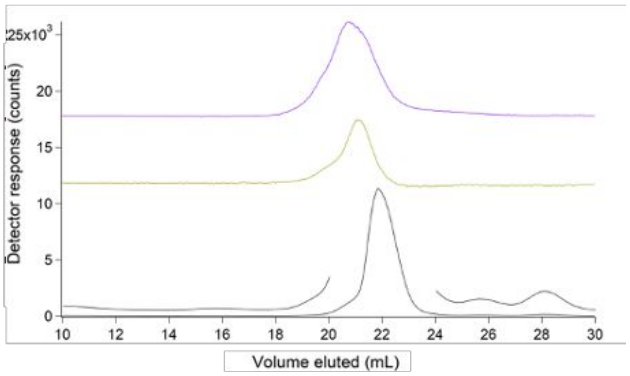

Supplement: Supplementary file 1 — Supplementary file1 (PDF 919 kb) [file 775_2021_1864_MOESM1_ESM.pdf]
